# Prediction of body weight and ethnicity using anthropomorphic measurements of the hand in two different populations

**DOI:** 10.1038/s41598-026-43161-z

**Published:** 2026-04-04

**Authors:** Heba Abdel Samie Mohamed Hussein, Omneya Ibrahim Mohamed, Rasha Ismail Khedr

**Affiliations:** Department of Forensic Medicine and Clinical Toxicology, Faculty of Medicine, Alexandria University, Alexandria, Egypt

**Keywords:** Body mass, Ancestry, Palm, Phalanges, Anthropometry, Identification, Egyptians, Saudis, Anatomy, Health care, Medical research

## Abstract

This study aimed to evaluate the usefulness of hand and finger measurements for prredicting body weight and distinguishing between Egyptians and Saudis. Also, to design regression models to predict body weight from hand and finger measurements. A descriptive cross-sectional study was conducted among 160 adult volunteers from two population groups (80 Egyptians and 80 Saudis), aged 20 to 27. The measurements taken were body weight and anthropometric measurements of the right hand, which comprised the hand length, handbreadth, palm length, and total and phalangeal finger length. Univariate and multivariate linear regression and ROC curve analysis were performed in the statistical analyses. Egyptians had no statistically significantly bigger hands than the Saudis. Egyptian and Saudi male participants had statistically significantly larger hand measurements than their corresponding female. Various hand and finger measurements were strong predictors of body weight and could differentiate Egyptian and Saudi ethnicities with sufficient accuracy. The highest precision was observed in Saudi females, where the little finger measurement yielded the most accurate model (R^2^ = 0.817, SEE = 3.15 kg). Egyptian males showed a moderate predictive capacity (R2 = 0.634, SEE = 7.14 kg), heavily influenced by handbreadth, while Egyptian females relied on index and middle finger phalanges to achieve a model with R^2^ = 0.525. Saudi males presented the least precise multivariate results with an R^2^ = 0.553 and the highest margin of error (SEE = 14.453 kg). The integration of multiple linear regression improved estimation accuracy compared to univariate models, which generally exhibited higher error ranges between 9.13 kg and 11.33 kg. The index proximal phalangeal length (AUC = 0.715) has the most potent discriminatory ability to distinguish between Saudi and Egyptian males (sensitivity 85.0%, cut-off ≤ 2.6). For females, Handbreadth was the best ethnic discriminator with an Area under the Curve (AUC) of 0.818 (sensitivity 75.0%, cut-off ≤ 7.8). Anthropometric measurements of the hand and fingers could be used to predict weight and ethnicity in both Egyptian and Saudi populations. This may draw attention to the anticipated practical medico-legal identification.

**Supplementary Information:**

The online version contains supplementary material available at 10.1038/s41598-026-43161-z.

## Introduction

Anthropometry, when utilized in the forensic medico-legal field, is a comprehensive tool for distinguishing remains. It facilitates the estimation of age at death, sex, ethnicity, stature, and body weight, and identifies individualizing features such as amputations, fractures, ankyloses, deformities, and bone pathologies. Furthermore, it can potentially infer the cause of death. This comprehensive approach enables law enforcement agencies to achieve the primary objective of personal identification^1–3^.

Hand anthropometry, the systematic evaluation of hand dimensions, morphology, and proportions, is a powerful tool in forensic identification. It directly or indirectly indicates hand development and correlates with age group, sex, race, ethnicity, body mass index, nutritional status, and sedentary behavior. This wealth of information has practical applications in resolving legal, medical, and forensic issues, making it an indispensable resource for professionals in these fields^4–6^.

Since some anthropometric measurements, such as those of the hand and foot, can be valuable in predicting human sex, stature, age, and even ethnicity, it was assumed that body weight could also be predicted in this manner^7^.

Body weight is a fundamental part of human biology that can be used for identification in forensic investigations^8^. Estimating human body weight draws interest in many fields. It is used to assess growth patterns, adjust ideal dosages in therapeutic instances, and identify individuals in criminal cases and mass disasters^9^.

The classic method of body weight estimation typically uses a body weight scale that works well in everyday life^10^. Still, in forensic cases, it might be hard enough to do so in cases of dead bodies, especially when they become disintegrated into pieces in mass disasters. In addition, there may be a difficulty that physicians face in calculating the dosage of some drugs for bedridden people and patients in the intensive care unit who do not know their body weights^11^. These scenarios mandate the development of proxy-based methods to estimate body weight from fragmented remains rather than relying on a whole-body weight scale.

However, several factors can influence the accuracy of these predictions, with ethnic differences playing a notable role. Anthropometric measurements demonstrate ethnic disparities between communities and are influenced by genetic and environmental factors, nutrition, sex, age, and physical activity^12–14^.

Despite the growing body of literature on anthropometric estimation methods and their use in stature estimation, its application for body weight prediction is under-researched, especially in Middle Eastern populations.

While previous studies have successfully utilized hand dimensions to estimate stature and sex across various global populations, the predictive power of hand anthropometry for body weight remains poorly defined. Furthermore, existing regression models are often population-specific; models derived from Western or East Asian cohorts may not accurately reflect the unique morphological characteristics of Middle Eastern populations, such as Egyptians and Saudis^15^. These groups, while geographically proximate, possess distinct genetic and cultural ancestries that are reflected in their physical characteristics. Therefore, a direct comparison of their anthropometric profiles is essential for forensic and clinical applications within the region.

We hypothesized that specific hand or individual finger dimensions would show a significant correlation with body weight in both Egyptians and Saudis, allowing for the creation of population-specific regression equations. Additionally, we predicted that significant dimorphic and ethnic variations in hand proportions would allow for a statistically significant classification between Egyptian and Saudi individuals.

To date, there is a notable absence of comparative data specifically evaluating the reliability of hand dimensions including palm length and finger dimensions for weight estimation within and between Egyptian and Saudi populations. Our study, which incorporates palm length into hand anthropometry, aims to fill this gap. The findings of our study could have significant implications for the fields of anthropology and health sciences, as we aimed to determine body weight from hand dimensions in Egyptians and Saudis and to derive regression models for this determination. Also, to distinguish between the two populations based on their hand and finger anthropological measurements.

## Subjects and methods

*Study design*: A descriptive cross-sectional analysis.

### Sample size calculation

G*Power software was employed to calculate the required sample size, referencing data from comparable studies. The anticipated accuracy of anthropometric measurements for predicting body weight and population variance was estimated at 70%. A minimum of 60 participants was determined a priori to achieve adequate statistical power for multivariate linear regression. The analysis targeted an alpha level of 0.05 and a statistical power of 0.80. With an assumed medium effect size (f2 = 0.15), this sample size was calculated to detect significant predictors of body weight. This approach ensures that the models developed for the Egyptian and Saudi cohorts are statistically robust, capable of explaining a substantial proportion of weight variance (R2), and yield minimized Standard Error of Estimates (SEE)^16,17^.

### Sampling methodology

*Selection of samples*: A random sampling technique was utilized. Participants were selected using a random number generator applied to a pre-existing list of university students’ names. The sample comprised healthy adult volunteers aged 20 to 27 years from two population groups: 80 Egyptians and 80 Saudis^18^. The data for both the Egyptian and Saudi cohorts were collected at Alexandria University Faculty of Medicine. All measurements were conducted between September 2024 and June 2025.

*Criteria for exclusion*: Subjects exhibiting hand deformities, diseases, traumas, congenital abnormalities, growth anomalies, or having undergone hand surgeries were excluded from the study^19^.

### Data acquisition

Each participant’s informed consent was obtained, and a comprehensive set of demographic data was gathered through a straightforward questionnaire. This thorough process guaranteed the completeness and accuracy of the data collected.

### Data acquisition methodology

The subsequent measurements were obtained for the right hand of each participant to the closest centimeter utilizing a digital Vernier calliper and a measuring tape as necessary. The measurements adhered to the protocols established by Davies et al.^20^, Courtney and Ng^21^, , Kemper and Schwerdtfeger^22^, and Imrhan et al.^23^.

Each participating subject positioned their hand in a supinated orientation with fully extended fingers (not hyperextended). All measurements were conducted on the subjects’ right hands, as negligible variations were seen between the right and left hands^24,25^. The dimensions of the hand are not influenced by hand laterality^19,26–28^.

### Anthropometric measurements

These comprised hand measurements, finger measurements, and body weight.

**Fig. 1:**
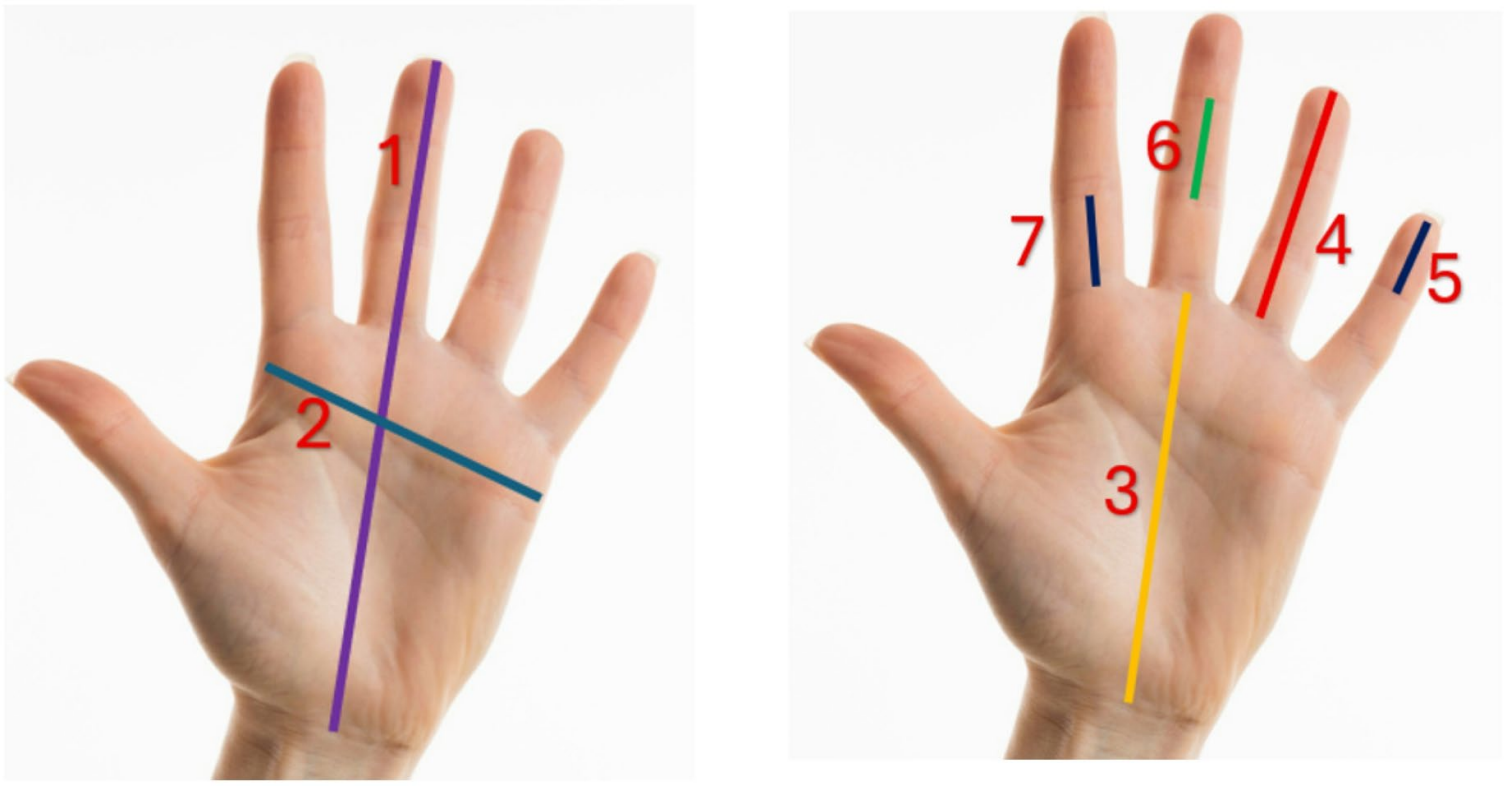
Hand and finger measurements, 1: Measurement of Hand Length, 2: Measurement of Handbreadth, 3: Measurement of palm Length, 4: Measurement of Total Finger Length, 5: Measurement of Distal Phalanx Length, 6: Measurement of Middle Phalanx Length, 7: Measurement of Proximal Phalanx Length (Proximal Phalanx L).

1. Hand measurements (Fig. 1).
  A. *Hand Length (HL)*: The measurement from the inter-stylion’s midpoint to the middle finger’s tip. Inter-stylion is the midpoint of the line connecting the stylion radial (the most distal point on the styloid process of the radius) and the stylion ulnare (the most distal point on the styloid process of the ulna).
  B. *Hand breadth*: The measurement from the metacarpal radiale (the most prominent external point of the lower epiphysis of the second metacarpal) to the metacarpal ulnare (the most prominent internal point of the lower epiphysis of the fifth metacarpal)^29^.
  C. *Palm Length*: The palm length is the differential measurement between hand length and middle finger length.
2. Finger measurements (Fig. 1).

The entire Length of each finger and the lengths of the distal and proximal phalanges were measured. The Length of the middle phalanx was measured in all fingers, excluding the thumb^19,30–32^.

A. *Total finger length*: the measurement from the proximal flexion crease of the finger to the tip of the corresponding digit.
B. *Distal phalange length*: the measurement from the most anteriorly protruding point on the tip of each finger to the distal interphalangeal joint crease of each finger.
C. *Length of the middle phalanx*: the measurement from the distal interphalangeal joint crease to the proximal interphalangeal joint crease.
D. *Proximal phalange length*: the measurement from the proximal interphalangeal joint crease to the metacarpophalangeal joint crease of each digit.

3. Body weight:

Participants were instructed to remove their heavy outer garments and footwear before weight measurement. The electronic scale recorded the weight according to standard protocols^33^.

### Reliability of the measurements

To assess measurement reliability, 20 subjects were randomly selected to repeat anthropometric measurements two weeks after the initial evaluation by the same investigator, thereby determining test-retest reliability. Simultaneously, a different investigator assessed 30 subjects to evaluate inter-examiner reliability. Quantitative metrics included the Intraclass Correlation Coefficient (ICC) with 95% Confidence Intervals (CI), Technical Error of Measurement (TEM), relative Technical Error of Measurement (rTEM), and Coefficient of Variation (CV%). The overall test-retest and inter-examiner reliability were excellent (ICC > 0.9). The reliability testing was a validation step performed on a random subset of participants (*n* = 30 for inter-observer and *n* = 20 for intra-observer), while the main comparative and predictive analyses utilized the first set of measurements from the full cohort (*N* = 160)^34,35^.

### Ethical considerations

The Research Ethics Committee of the Faculty of Medicine at Alexandria University approved the study (IRB number: 00012098, serial number: 0306926). Informed consent was obtained from all participants before their involvement in the study. The confidentiality of the patient’s data was taken into account. At the same time, all methods were accomplished following the Declaration of Helsinki.

### Analysis of the data using statistical methods

The data was input into the computer and analyzed utilizing IBM SPSS version 20.0. IBM Corp, Armonk, NY: Released 2011. The Shapiro-Wilk test was initially applied to all continuous variables; including age, body weight, hand length, handbreadth, and all finger phalangeal lengths to assess the normality of the distribution. Categorical data, such as sex and ethnicity, were expressed as frequencies and percentages and compared using the Chi-square test. To compare mean differences in age, body weight, and hand metrics between the two nationalities (Egyptian vs. Saudi), the Independent t-test was utilized. For comparisons involving more than two groups, specifically when analyzing hand and finger measurements across the four sex-and-ethnicity subgroups, one-way ANOVA (F-test) was employed, followed by the Tukey Post Hoc test for significant pairwise comparisons. The relationship between hand dimensions, finger lengths and body weight was evaluated using the Pearson correlation coefficient (r). Subsequently, linear regression analysis was performed with body weight as the dependent variable and hand and finger metrics as independent predictors. To account for the confounding effects of ethnicity and biological sex on body weight, the study population was stratified into four distinct groups (Egyptian males, Egyptian females, Saudi males, and Saudi females). Separate univariate and multivariate regression models were then constructed for each group to ensure the precision of body weight predictions independent of these factors.

*Multicollinearity and Model Parsimony*: To ensure the stability of the multiple linear regression coefficients, multicollinearity diagnostics were performed using the Variance Inflation Factor (VIF). Variables were screened for inclusion based on their biological relevance and individual correlation with body weight. In the multivariate models, any predictor exhibiting a VIF > 3.0 was considered for exclusion to prevent over-inflation of the coefficient of determination (R^2^) and to ensure the stability of the regression coefficients. This threshold ensures that the variance-covariance structure of the hand measurements did not adversely affect the precision of the weight predictions. Supplementary File 1.

*Regression Diagnostics and Assumption Testing* Prior to the construction of the regression equations, the fundamental assumptions of linear regression were rigorously tested. Normality of the data distribution and residuals was assessed using the Shapiro-Wilk test and visual inspection of Q-Q plots. Homoscedasticity (homogeneity of variance) was verified by plotting studentized residuals against predicted values to ensure a random distribution. To identify influential outliers that might skew the results, Cook’s Distance (D) was calculated for all subjects; no participant exceeded the threshold of D > 1, and thus all 80 subjects were retained for analysis. Supplementary File 2.

Receiver Operating Characteristic (ROC) curves were used to determine the sensitivity and specificity of hand measurements in differentiating male and female Saudis from Egyptians, with 95% Confidence Intervals (CI) for the Area Under the Curve (AUC) calculated via the DeLong method. The Youden index was employed to ascertain the optimal cut-off value. The Youden index represents the vertical distance between the 45-degree line and a certain point on the ROC curve. Sensitivity, specificity, and accuracy were computed. The significance was assessed at the 5% threshold^36,37^.

## Results

This study analyzed 160 individuals: 80 Egyptians (mean age 23.59 ± 1.54) and 80 Saudis (mean age 23.20 ± 1.21), with no significant age difference (t = 1.772, *p* = 0.078). Egyptians had a higher mean body weight (76.46 ± 12.49 kg), but the difference was not significant (t = 1.761, *p* = 0.081) (Table 1).

**Table 1:** Comparison between the participating Egyptians and Saudis according to age, sex and body weight. (*n* = 160)

	Egyptians (*n* = 80)	Saudis (*n* = 80)	Significance Test	*p*
Age (years)				
Min. – Max.	20–27	22–27	t = 1.772	0.078
Mean ± SD.	23.59 ± 1.54	23.20 ± 1.21		
Sex				
Females	40 (50.0%)	40 (50.0%)	χ^2^ = 0.000	1.000
Males	40 (50.0%)	40 (50.0%)		
Body weight				
Min. – Max.	40–99	50–135	t = 1.761	0.081
Mean ± SD.	76.46 ± 12.49	71.32 ± 22.92		

SD: Standard deviation t: Student t-test.

χ^2^: Chi-square test.

p_1_: p-value for comparing Egyptians and Saudis.

### Measurement reliability and reproducibility

The results of the reliability assessment for all hand and finger measurements are summarized in Table 2. The inter-observer reliability (*n* = 30) demonstrated excellent consistency, with Intraclass Correlation Coefficients (ICC) ranging from 0.983 to 1.000 and Technical Error of Measurement (TEM) values consistently below 0.183. Similarly, the intra-observer (test-retest) reliability (*n* = 20) yielded high reproducibility, with ICCs ranging from 0.911 to 0.990. The low magnitude of the TEM and Coefficient of Variation (CV%) across all parameters confirms that measurement error was negligible and that the anthropometric landmarks were identified consistently by different investigators and across different time points denoting excellent inter-rater and intra-rater reliability. At the same time, Inter-observer relative Technical Error of Measurement (rTEM%) values ranged from 0.00% to 1.65%, while intra-observer rTEM% ranged from 0.05% to 1.21%.

**Table 2:** Reliability metrics of all the primary measurements.

		Inter-rater (*n* = 30)				Intra-rater(test–retest) (*n* = 20)			
		ICC (95% C.I.)	TEM	rTEM%	CV%	ICC (95% C.I.)	TEM	rTEM%	CV%
Hand	Length	0.988 (0.975–0.994)	0.183	0.986	9.0	0.989 (0.972–0.995)	0.192	1.017	9.7
	Breadth	0.995 (0.990–0.998)	0.063	0.771	10.9	0.990 (0.975–0.996)	0.130	1.200	15.0
	Palm length	0.987 (0.974–0.994)	0.124	1.197	10.5	0.985 (0.964–0.994)	0.122	1.188	9.7
Thumb	Total length	0.983 (0.965–0.992)	0.100	1.486	11.4	0.982 (0.955–0.993)	0.114	1.077	12.5
	Distal phalangeal length	1.000 (–)	0.000	0.000	13.7	0.971 (0.925–0.989)	0.110	1.116	18.3
	Proximal phalangeal length	0.998 (0.997–0.999)	0.026	0.868	19.4	0.971 (0.775–0.992)	0.100	1.014	17.7
Index	Total length	0.994 (0.987–0.997)	0.052	0.713	9.2	0.976 (0.929–0.991)	0.105	1.164	9.0
	Distal phalangeal length	1.000 (–)	0.000	0.000	14.0	0.926 (0.774–0.973)	0.095	0.755	12.7
	Middle phalangeal length	1.000 (–)	0.000	0.000	9.9	0.949 (0.841–0.939)	0.100	1.080	10.5
	Proximal phalangeal length	1.000 (–)	0.000	0.000	18.6	0.911 (0.888–0.974)	0.100	0.058	13.4
Middle	Total length	0.985 (0.969–0.993)	0.097	1.237	10.1	0.989 (0.924–0.997)	0.095	1.154	11.0
	Distal phalangeal length	1.000 (–)	0.000	0.000	14.4	0.958 (0.955–0.987)	0.095	0.484	17.0
	Middle phalangeal length	0.993 (0.984–0.996)	0.037	1.339	16.0	0.929 (0.956–0.979)	0.100	0.364	13.0
	Proximal phalangeal length	0.996 (0.991–0.998)	0.026	0.917	14.5	0.925 (0.910–0.973)	0.100	0.442	13.3
Ring	Total length	0.989 (0.976–0.995)	0.086	1.175	11.2	0.984 (0.976–0.996)	0.110	1.205	11.9
	Distal phalangeal length	0.987 (0.972–0.994)	0.045	1.653	14.5	0.929 (0.927–0.978)	0.095	0.544	13.3
	Middle phalangeal length	1.000 (–)	0.000	0.000	16.0	0.949 (0.940–0.985)	0.100	0.607	17.3
	Proximal phalangeal length	1.000 (–)	0.000	0.000	22.2	0.948 (0.908–0.984)	0.095	0.196	18.4
Little	Total length	0.991 (0.981–0.996)	0.063	1.081	11.4	0.988 (0.917–0.996)	0.095	1.166	14.3
	Distal phalangeal length	1.000 (–)	0.000	0.000	15.0	0.938 (0.898–0.982)	0.100	1.108	16.5
	Middle phalangeal length	1.000 (–)	0.000	0.000	16.5	0.931 (0.889–0.952)	0.110	0.779	14.3
	Proximal phalangeal length	1.000 (–)	0.000	0.000	29.5	0.963 (0.929–0.990)	0.100	0.751	29.9
Overall		1.000 (1.000–1.000)	0.064	0.000	81.5	1.000 (1.000–1.000)	0.109	0.000	80.8

### Sex and ethnicity regarding hand and individual finger measurements

Table 3 shows that Egyptian and Saudi males had significantly higher hand length, palm length, handbreadth, and thumb phalangeal lengths than females (p1 < 0.001, p2 < 0.001).

**Table 3:** Comparison between the participated Egyptians and Saudis according to hand and thumb measurements (*n* = 160).

	Hand Measurements	Egyptians (*n* = 80)		Saudis (*n* = 80)		Test of Significance (p)
		Males (*n* = 40)	Females (*n* = 40)	Males (*n* = 40)	Females (*n* = 40)	
Hand	Length					
	Min. – Max.	18.2–21	16.5–19	18.7–22	16–18	p_1_<0.001^*^, p_2_ < 0.001^*^, p_3_ = 0.939, p_4_ = 0.001^*^
	Mean ± SD.	20.11 ± 1.12	17.54 ± 0.84	19.99 ± 0.86	16.75 ± 0.59	
	Breadth					
	Min. – Max.	8.50–10.3	7–9	8.80–11	7–8	p_1_<0.001^*^, p_2_ < 0.001^*^, p_3_ < 0.001^*^, p_4_ < 0.001^*^
	Mean ± SD.	9.30 ± 0.41	8.02 ± 0.47	9.84 ± 0.70	7.45 ± 0.38	
	Palm length					
	Min. – Max.	10.3–12.7	8.50–11.5	10.2–12.8	9–10	p_1_<0.001^*^, p_2_ < 0.001^*^, p_3_ = 0.738, p_4_ = 0.272
	Mean ± SD.	11.41 ± 0.88	9.85 ± 0.73	11.26 ± 0.57	9.57 ± 0.46	
Thumb	Total length					
	Min. – Max.	6.90–8	5.50–7.50	6.50–8.80	5–6.50	p_1_<0.001^*^, p_2_ < 0.001^*^, p_3_= 1.000, p_4_ = 0.003^*^
	Mean ± SD.	7.38 ± 0.41	6.36 ± 0.53	7.38 ± 0.55	5.99 ± 0.38	
	Distal phalangeal length					
	Min. – Max.	3.20–4.30	2.50–4	3.50–4.50	2.50–3.50	p_1_<0.001^*^, p_2_ < 0.001^*^, p_3_ = 0.991, p_4_ = 0.925
	Mean ± SD.	3.94 ± 0.33	3.08 ± 0.42	3.96 ± 0.29	3.13 ± 0.31	
	Proximal phalangeal length					
	Min. – Max.	2.60–4.60	2.40–4	2.50–4.80	2.50–3.50	p_1_=0.482, p_2_ < 0.001^*^, p_3_ = 0.998, p_4_ = 0.002^*^
	Mean ± SD.	3.44 ± 0.64	3.28 ± 0.45	3.42 ± 0.56	2.86 ± 0.37	

SD: Standard deviation.

Pairwise comparison bet. each 2 groups was done using Post Hoc Test (Tukey) for One way ANOVA test.

p_1_: p value for comparing between Males Egyptians and Females Egyptians.

p_2_: p value for comparing between Males Saudis and Females Saudis.

p_3_: p value for comparing between Males Egyptians and Males Saudis.

p_4_: p value for comparing between Females Egyptians and Females Saudis.

*: Statistically significant at *p* < 0.05.

Among males, there was no significant difference in hand length (20.11 ± 1.12 vs. 20.11 ± 1.12, p3 = 0.939) or palm length (11.41 ± 0.88, p3 = 0.738) between groups, though Egyptians had slightly higher means. Saudi males had significantly larger handbreadth (P3 < 0.001). No significant differences were seen in total or individual thumb phalangeal lengths (p3 = 1.000, 0.991, 0.998).

Egyptian females had higher hand length (17.54 ± 0.84), handbreadth (8.02 ± 0.47), palm length (9.85 ± 0.73), and total thumb length (6.36 ± 0.53) than Saudis. All differences were significant except palm length (*p* = 0.272) and distal thumb phalanx (*p* = 0.925). Egyptian females showed significantly greater hand length, handbreadth, total finger, and proximal thumb phalanx (*p* = 0.001, < 0.001, 0.003, 0.002) (Table 3).

Table 4 compares Egyptian and Saudi participants according to other fingers’ total and individual phalangeal lengths. Male participants from both populations had statistically significantly higher index, ring, middle, and little finger measurements than females.

**Table 4:** Comparison between the participated Egyptians and Saudis according to individual finger measurements (*n* = 160).

	Hand Measurements	Egyptians (*n* = 80)		Saudis (*n* = 80)		Test of Significance (p)
		Males (*n* = 40)	Females (*n* = 40)	Males (*n* = 40)	Females (*n* = 40)	
Index	Total length					
	Min. – Max.	7–8.80	6–8	7–8.50	6.10–7.50	p_1_<0.001^*^, p_2_ < 0.001^*^, p_3_ = 0.762, p_4_ < 0.001^*^
	Mean ± SD.	7.89 ± 0.57	7.16 ± 0.47	7.79 ± 0.42	6.59 ± 0.33	
	Distal phalangeal length					
	Min. – Max.	2.40–3.60	2–3	2.50–3.30	2–2.50	p_1_<0.001^*^, p_2_ < 0.001^*^, p_3_ = 0.207, p_4_ = 0.796
	Mean ± SD.	2.89 ± 0.28	2.40 ± 0.36	3 ± 0.16	2.35 ± 0.16	
	Middle phalangeal length					
	Min. – Max.	2–2.60	2–2.50	2.20–2.80	2–2.50	p_1_=0.338, p_2_ < 0.001^*^, p_3_ = 0.204, p_4_ < 0.001^*^
	Mean ± SD.	2.39 ± 0.20	2.32 ± 0.22	2.48 ± 0.15	2.09 ± 0.19	
	Proximal phalangeal length					
	Min. – Max.	2–3	1.70–3	1.60–2.90	2–2.60	p_1_=0.090, p_2_ = 0.105, p_3_ < 0.001^*^, p_4_ = 0.001^*^
	Mean ± SD.	2.61 ± 0.35	2.44 ± 0.39	2.32 ± 0.34	2.15 ± 0.19	
Middle	Total length					
	Min. – Max.	7.90–9.50	6.50–9	7.50–9.50	7–8.70	p_1_<0.001^*^, p_2_ < 0.001^*^, p_3_ = 0.985, p_4_ < 0.001^*^
	Mean ± SD.	8.70 ± 0.57	7.69 ± 0.61	8.74 ± 0.50	7.18 ± 0.46	
	Distal phalangeal length					
	Min. – Max.	2.20–3.40	1.50–3	2.50–3.50	1.80–2.70	p_1_<0.001^*^, p_2_ < 0.001^*^, p_3_ = 0.003^*^, p_4_ = 0.278
	Mean ± SD.	2.90 ± 0.35	2.43 ± 0.34	3.13 ± 0.20	2.31 ± 0.26	
	Middle phalangeal length					
	Min. – Max.	2.30–3.80	2.40–3.50	2.30–3.10	2.20–3	p_1_=0.005^*^, p_2_ < 0.001^*^, p_3_ = 0.900, p_4_ < 0.001^*^
	Mean ± SD.	2.93 ± 0.46	2.68 ± 0.30	2.88 ± 0.19	2.38 ± 0.29	
	Proximal phalangeal length					
	Min. – Max.	2.30–3.50	2–3.40	2.10–3.40	2.30–3	p_1_=0.002^*^, p_2_ = 0.011^*^, p_3_ = 0.326, p_4_ = 0.643
	Mean ± SD.	2.88 ± 0.42	2.58 ± 0.37	2.74 ± 0.41	2.49 ± 0.20	
Ring	Total length					
	Min. – Max.	7.40–8.70	6–8	6.70–9	6–7.70	p_1_<0.001^*^, p_2_ < 0.001^*^, p_3_ = 0.878, p_4_ < 0.001^*^
	Mean ± SD.	7.94 ± 0.35	6.93 ± 0.63	8.02 ± 0.46	6.33 ± 0.50	
	Distal phalangeal length					
	Min. – Max.	1.90–3.20	2–3.60	2.50–3.60	2–2.70	p_1_=0.121, p_2_ < 0.001^*^, p_3_ = 0.001^*^, p_4_ = 0.068
	Mean ± SD.	2.69 ± 0.42	2.54 ± 0.37	2.98 ± 0.23	2.36 ± 0.19	
	Middle phalangeal length					
	Min. – Max.	2.50–3.50	2–2.60	2.20–3	1.90–2.90	p_1_<0.001^*^, p_2_ < 0.001^*^, p_3_ < 0.001^*^, p_4_ = 0.762
	Mean ± SD.	2.96 ± 0.36	2.27 ± 0.23	2.61 ± 0.21	2.21 ± 0.28	
	Proximal phalangeal length					
	Min. – Max.	2–3	1–3	1.20–3.20	1.60–2.10	p_1_=0.172, p_2_ < 0.001^*^, p_3_ = 0.302, p_4_ < 0.001^*^
	Mean ± SD.	2.29 ± 0.27	2.12 ± 0.52	2.43 ± 0.40	1.76 ± 0.17	
Little	Total length					
	Min. – Max.	5.50–7.70	5–6.50	5.50–7.60	5–6	p_1_<0.001^*^, p_2_ < 0.001^*^, p_3_ = 0.007^*^, p_4_ = 0.003^*^
	Mean ± SD.	6.39 ± 0.61	5.51 ± 0.51	6.75 ± 0.53	5.11 ± 0.29	
	Distal phalangeal length					
	Min. – Max.	1.50–4.20	1.50–2.60	2.40–3	2–2.50	p_1_=0.001^*^, p_2_ < 0.001^*^, p_3_ = 0.002^*^, p_4_ = 0.852
	Mean ± SD.	2.51 ± 0.62	2.19 ± 0.27	2.81 ± 0.19	2.25 ± 0.16	
	Middle phalangeal length					
	Min. – Max.	1.50–2.20	1–2.50	1.50–2.10	1.40–2	p_1_=0.001^*^, p_2_ < 0.001^*^, p_3_ = 0.173, p_4_ = 0.998
	Mean ± SD.	1.86 ± 0.26	1.64 ± 0.34	1.97 ± 0.14	1.63 ± 0.19	
	Proximal phalangeal length					
	Min. – Max.	1.50–3	1.30–3	1–2.70	1–2	p_1_=0.002^*^, p_2_ < 0.001^*^, p_3_ = 0.965, p_4_ < 0.001^*^
	Mean ± SD.	2.02 ± 0.47	1.69 ± 0.40	1.98 ± 0.42	1.24 ± 0.29	

SD: Standard deviation, Pairwise comparison bet. Each 2 groups was done using Post Hoc Test (Tukey) for One way ANOVA test.

p1: p-value for comparing Male Egyptians and Female Egyptians.

p2: p-value for comparing Male Saudis and Female Saudis.

p3: p-value for comparing between Males Egyptians and Males Saudis.

p4: p-value for comparing Egyptian females and Saudi females *: Statistically significant at *p* < 0.05.

In males, Egyptians had higher proximal index phalangeal (*p* < 0.001) and ring middle phalangeal (*p* < 0.001) lengths. Saudis had higher distal middle (*p* = 0.003), distal ring (*p* = 0.001), and both total and distal little finger lengths (*p* = 0.007, 0.002) (Table 4).

Table 4 depicts that Egyptian females had higher individual finger measurements than Saudi females, except for the distal phalanx of the little finger. Significant differences were found in total, middle, and proximal phalanges of the index, middle, ring, and little fingers (*p* < 0.001, 0.003).

### Prediction of body weight using univariate and multivariate linear regression analysis of hand and individual finger measurements

Table 5 demonstrates that in Egyptian males, body weight was significantly correlated with hand and finger measurements. The middle phalangeal length of the index finger had the strongest negative correlation (*r*= -0.552), followed by handbreadth (*r* = 0.519).

**Table 5:** Univariate linear regression analysis to predict body weight from all the studied hand and fingers’ measurements of Egyptian males and females (*n* = 80).

	Egyptian males (*n* = 40)					Egyptian females (*n* = 40)				
	*r*	Univariate			VIF	*r*	Univariate			VIF
		*p*	SEE	B (95%C. I)			*p*	SEE	B (95%C. I)	
Hand										
Length	-0.424^*^	0.006^*^	10.264	-4.253 (-7.236 – -1.269)	1.829	0.140	0.388	11.034	1.846 (-2.436–6.128)	
Breadth	0.519^*^	0.001^*^	9.687	14.145 (6.494–21.796)	1.081	0.047	0.773	11.132	1.099 (-6.566–8.764)	
Palm length	-0.308	0.053	10.782	-3.911 (-7.879–0.058)		-0.153	0.345	11.012	-2.303 (-7.178–2.571)	
Thumb										
Total length	-0.118	0.470	11.254	-3.194 (-12.046–5.658)		0.089	0.585	11.100	1.849 (-4.939–8.637)	
Distal phalangeal length	-0.076	0.639	11.300	-2.561 (-13.524–8.402)		-0.065	0.689	11.121	-1.715 (-10.332–6.903)	
Proximal phalangeal length	-0.036	0.827	11.326	-0.623 (-6.345–5.098)		0.167	0.302	10.987	4.135 (-3.865–12.135)	
Index										
Total length	-0.508^*^	0.001^*^	9.763	-9.993 (-15.560 – -4.426)		0.153	0.347	11.013	3.575 (-4.021–11.170)	
Distal phalangeal length	-0.390^*^	0.013^*^	10.434	-15.608 (-27.701 – -3.515)	1.258	0.572^*^	< 0.001^*^	9.138	17.329 (9.177–25.480)	1.383
Middle phalangeal length	-0.552^*^	< 0.001^*^	9.453	-30.501 (-45.649 – -15.354)		0.281	0.079	10.696	13.863 (-1.692–29.417)	
Proximal phalangeal length	-0.192	0.234	11.121	-6.096 (-16.304–4.112)		-0.513^*^	0.001^*^	9.565	-14.566 (-22.568 – -6.564)	1.110
Middle										
Total length	-0.356^*^	0.024^*^	10.589	-7.047 (-13.115 – -0.979)	2.011	0.376^*^	0.017^*^	10.328	6.767 (1.284–12.249)	1.628
Distal phalangeal length	0.300	0.060	10.813	9.673 (-0.446–19.792)		0.366^*^	0.020^*^	10.373	11.788 (1.932–21.643)	1.475
Middle phalangeal length	-0.396^*^	0.011^*^	10.404	-9.723 (-17.116 – -2.329)		0.205	0.203	10.906	7.520 (-4.242–19.281)	
Proximal phalangeal length	-0.297	0.062	10.820	-7.944 (-16.323–0.434)		0.116	0.476	11.069	3.456 (-6.255–13.167)	
Ring										
Total length	0.001	0.996	11.333	0.025 (-10.365–10.414)		0.180	0.266	10.962	3.166 (-2.515–8.847)	
Distal phalangeal length	-0.279	0.081	10.881	-7.471 (-15.901–0.959)		0.279	0.082	10.703	8.321 (-1.099–17.740)	
Middle phalangeal length	0.411^*^	0.008^*^	10.333	12.935 (3.502–22.368)		0.209	0.195	10.898	-1.579 (-8.506–5.347)	
Proximal phalangeal length	-0.107	0.510	11.267	-4.496 (-18.186–9.195)		-0.075	0.647	11.113	9.863 (-5.274–25.001)	
Little										
Total length	-0.171	0.290	11.165	-3.149 (-9.092–2.794)		0.070	0.667	11.117	1.522 (-5.579–8.623)	
Distal phalangeal length	-0.267	0.096	10.922	-4.803 (-10.503–0.898)		0.175	0.280	10.972	7.078 (-6.002–20.159)	
Middle phalangeal length	-0.141	0.386	11.220	-6.040 (-19.969–7.889)		-0.005	0.978	11.144	-0.146 (-10.653–10.360)	
Proximal phalangeal length	0.208	0.197	11.084	4.947 (-2.685–12.579)		-0.026	0.873	11.140	-0.728 (-9.847–8.392)	

B: Unstandardized Coefficients C.I: Confidence interval LL: Lower limit UL: Upper limit SEE: Standard error of estimate.

*: Statistically significant at *p* ≤ 0.05.

For Egyptian females, distal and proximal index phalangeal lengths, total and distal middle finger lengths predicted body weight. The distal index phalanx was the most significant predictor (B = 17.329) (Table 5).

For Saudi males, body weight predictors included handbreadth, thumb distal phalanx, index total and middle phalanges, and middle finger total length. Handbreadth (*r* = 0.621) and index middle phalanx (*r* = 0.533) were most significant (Table 6).

**Table 6:** Univariate linear regression analysis to predict body weight from all the studied hand and fingers’ measurements of Saudi males and females (*n* = 80).

	Saudi males (*n* = 40)					Saudi females (*n* = 40)				
	*r*	Univariate			VIF	*r*	Univariate			VIF
		*p*	SEE	B (95%C. I)			*p*	SEE	B (95%C. I)	
Hand										
Length	0.029	0.859	20.731	0.686 (-7.085–8.457)		0.781^*^	< 0.001^*^	4.477	9.407 (6.941–11.874)	
Breadth	0.621^*^	< 0.001^*^	16.262	18.215 (10.657–25.772)	1.467	0.593^*^	< 0.001^*^	5.775	10.995 (6.098–15.892)	1.000
Palm length	-0.245	0.128	20.110	-8.802 (-20.262–2.658)		0.037	0.819	7.170	0.580 (-4.502–5.661)	
Thumb										
Total length	0.054	0.738	20.709	2.044 (-10.256–14.343)		0.322^*^	0.043^*^	6.793	5.946 (0.208–11.683)	
Distal phalangeal length	0.404^*^	0.010^*^	18.972	28.366 (7.275–49.456)	1.898	-0.353^*^	0.025^*^	6.713	-8.100 (-15.146 – -1.054)	1.160
Proximal phalangeal length	-0.157	0.334	20.484	-5.703 (-17.512–6.106)		0.629^*^	< 0.001^*^	5.577	12.046 (7.159–16.934)	1.160
Index										
Total length	0.320^*^	0.044^*^	19.647	15.474 (0.445–30.503)	2.680	0.774^*^	< 0.001^*^	4.546	16.405 (11.993–20.818)	
Distal phalangeal length	0.199	0.219	20.326	25.288 (-15.660–66.237)		0.109	0.505	7.133	4.712 (-9.448–18.871)	
Middle phalangeal length	0.533^*^	< 0.001^*^	17.550	74.551 (35.670–113.431)	1.681	0.695^*^	< 0.001^*^	5.157	25.593 (16.904–34.282)	1.074
Proximal phalangeal length	0.076	0.643	20.680	4.544 (-15.123–24.212)		0.549^*^	< 0.001^*^	5.998	19.956 (9.974–29.938)	1.074
Middle										
Total length	0.329^*^	0.038^*^	19.585	13.494 (0.772–26.215)		0.953^*^	< 0.001^*^	2.169	14.539 (13.025–16.053)	
Distal phalangeal length	0.162	0.319	20.467	16.559 (-16.668–49.787)		0.438^*^	0.005^*^	6.450	12.121 (3.953–20.288)	
Middle phalangeal length	0.309	0.052	19.722	33.251 (-0.313–66.815)		0.742^*^	< 0.001^*^	4.810	18.412 (12.950–23.875)	1.033
Proximal phalangeal length	0.177	0.275	20.413	8.758 (-7.262–24.779)		0.595^*^	< 0.001^*^	5.767	21.135 (11.761–30.508)	1.033
Ring										
Total length	0.148	0.363	20.513	6.522 (-7.832–20.875)		0.864^*^	< 0.001^*^	3.610	12.348 (9.987–14.708)	
Distal phalangeal length	0.065	0.692	20.696	5.695 (-23.150–34.540)		0.615^*^	< 0.001^*^	5.657	22.867 (13.244–32.491)	1.017
Middle phalangeal length	0.046	0.778	20.718	5.577 (-11.120–22.274)		0.681^*^	< 0.001^*^	5.253	29.912 (20.465–39.359)	1.017
Proximal phalangeal length	0.109	0.503	20.616	4.482 (-27.463–36.426)		0.721^*^	< 0.001^*^	4.974	17.466 (11.302–23.631)	
Little										
Total length	0.243	0.131	20.117	9.323 (-2.890–21.535)		0.868^*^	< 0.001^*^	3.562	21.318 (17.314–25.322)	
Distal phalangeal length	-0.032	0.844	20.729	-3.435 (-38.555–31.684)		0.419^*^	0.007^*^	6.514	18.932 (5.472–32.393)	1.243
Middle phalangeal length	0.084	0.608	20.667	12.377 (-36.006–60.760)		0.362^*^	0.022^*^	6.689	13.858 (2.139–25.577)	1.087
Proximal phalangeal length	0.293	0.066	19.827	14.168 (-0.992–29.328)		0.404^*^	0.010^*^	6.565	9.810 (2.506–17.114)	1.310

B: Unstandardized Coefficients C.I: Confidence interval LL: Lower limit UL: Upper Limit *: Statistically significant at *p* ≤ 0.05.

For Saudi females, all hand and finger measurements, except palm length and distal phalangeal length of the index finger, predicted body weight. Most influential were total middle (*r* = 0.953), little (*r* = 0.868), and ring (*r* = 0.864) finger lengths (Table 6).

Prediction accuracy improved using multivariate regression equations for each group. In male Egyptians’ model (R^2^ = 0.634), handbreadth is the strongest positive contributor here. The SEE is **7**.144 kg, which is a significant improvement over univariate models. In female Egyptians, the model relies heavily on the Index and Middle finger phalanges (R^2^ = 0.525).

On the other hand, the model of the Saudi males (R^2^ = 0.553) uses a mix of Handbreadth, Thumb distal phalangeal length, Index total length, and Middle phalangeal length. In Saudi females, six equations (hand and finger measurements) were all highly significant (*p* < 0.001), with R values from 0.593 to 0.904. For all the developed models, the standard error of estimates (SEEs) ranged from 3.156 for the little finger of the Saudi females (best performing model), to 14.453 for Saudi males (Table 7).

**Table 7:** Multiple linear regression equations for prediction of body weight from hand and fingers’ measurements of the two studied groups.

Population group	Gender	Equations	*R*	*R* ^2^	SEE	F-value	*p*	VIF range
Egyptians	Males	90.819–4.968*Hand Length + 14.734*Hand Breadth – 12.935*Index Distal phalangeal length − 0.955*Middle Total length	0.796	0.634	7.144	15.154^*^	< 0.001^*^	1.081–2.011
	Female	41.614 + 10.223 * Index Distal phalangeal length − 11.985*Index Proximal phalangeal length + 2.807*Middle Total length + 4.990 *Middle Distal phalangeal length	0.725	0.525	8.003	9.672^*^	< 0.001^*^	1.110–1.628
Saudis	Males	-133.025 + 16.706* Hand Breadth + 19.835*Thumb Distal phalangeal length − 22.134*Index total Length + 60.932*Middle phalangeal length	0.743	0.553	14.453	10.813	< 0.001^*^	1.467–2.680
	Female	Hand: -27.642 + 10.995*Hand Breadth	0.593	0.352	5.775	20.661^*^	< 0.001^*^	
		Thumb: 32.598–3.176*Thumb Distal phalangeal length + 11.061*Thumb Proximal phalangeal length	0.642	0.412	5.574	12.980^*^	< 0.001^*^	1.160
		Index: -21.908 + 21.791*Index Middle phalangeal length + 14.305*Index Proximal phalangeal length	0.792	0.627	4.438	31.161^*^	< 0.001^*^	1.074
		Middle: -14.711 + 9.480*Middle Middle phalangeal length + 18.959* Middle Proximal phalangeal length	0.684	0.468	5.305	16.260	< 0.001^*^	1.033
		Ring: -27.378 + 19.921* Ring Distal phalangeal length + 15.686* Ring Middle phalangeal length	0.864	0.746	3.661	54.468^*^	< 0.001^*^	1.017
		Little: -73.727 + 32.225*Little Distal phalangeal length + 18.396*Little Middle phalangeal length + 20.686*Little Proximal phalangeal length	0.904	0.817	3.156	53.454	< 0.001^*^	1.087–1.310

R^2^: Coefficient of determination SEE: Standard estimation error *: Statistically significant at *p* ≤ 0.05.

#: All variables with *p* < 0.05 were included in the multivariate analysis.

The precision of the univariate models (Table 5) yielded SEEs ranging from 9.13 to 11.33 kg, indicating that single-hand measurements explain a moderate portion of weight variance. By utilizing multiple linear regression (Table 7), the precision improved significantly. For Egyptian males, the multivariate model yielded an SEE of 7.14 kg, leaving 36.6% of the variance unexplained (100 - R^2^). The most precise model was observed in Saudi females using little finger measurements (R^2^ = 0.817, SEE = 3.15 kg), leaving only 18.3% of the total variance unexplained.

### Prediction of ethnicity from the hand and fingers’ measurements using ROC curve analysis

**Fig. 2:**
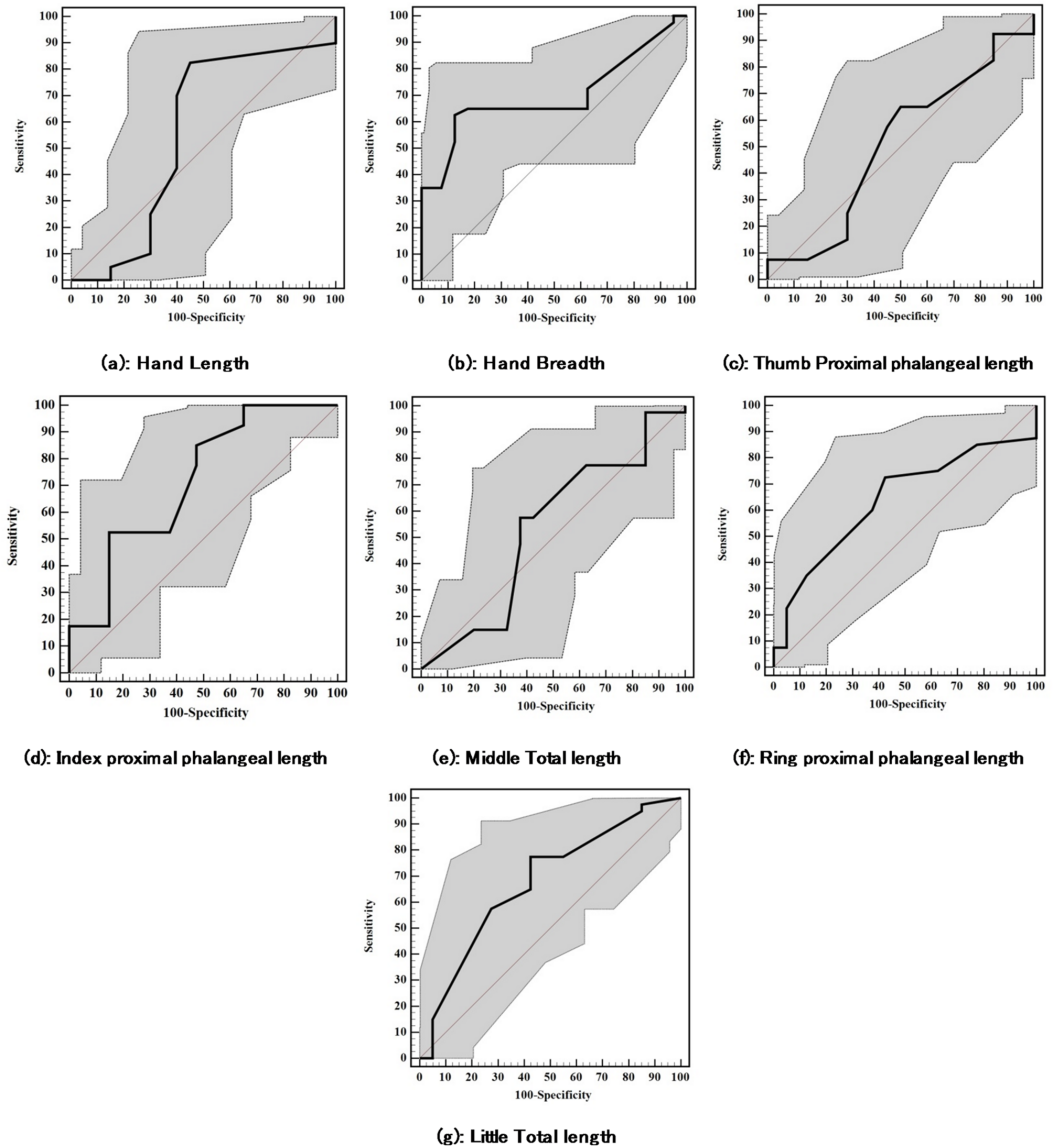
ROC curves for different measures to distinguish Saudis from Egyptians in male participants.

Table 8 and Fig. 2 show that for males, index proximal phalangeal length (AUC = 0.715) and handbreadth (AUC = 0.699) best distinguished between groups. Thumb proximal phalanx (AUC = 0.509) and middle total length (AUC = 0.534) had limited discrimination.

**Table 8:** Diagnostic performance for different measures to distinguish Saudis from Egyptians in male participants.

		AUC	*p*	95% C. I	Cut off	Sensitivity	Specificity	PPV	NPV	Accuracy
Hand	Length	0.558	0.376	0.422–0.693	≤ 20.5^#^	82.50	55.0	64.7	75.9	68.75
	Breadth	0.699	0.002^*^	0.578–0.819	> 9.3	65.0	67.50	66.67	65.85	66.25
Thumb	Proximal phalangeal length	0.509	0.893	0.380–0.638	> 3.2	65.0	50.0	56.52	58.82	57.50
Index	Proximal phalangeal length	0.715	0.001^*^	0.602–0.828	≤ 2.6	85.0	52.50	64.15	77.78	68.75
Middle	Total length	0.534	0.603	0.404–0.664	> 8.7^#^	57.50	62.50	60.53	59.52	60.0
Ring	Proximal phalangeal length	0.639	0.033^*^	0.514–0.763	> 2.2^#^	72.50	57.50	63.04	67.65	65.0
Little	Total length	0.677	0.006^*^	0.559–0.795	> 6.3^#^	77.50	57.50	64.58	71.88	67.50

AUC: Area Under a Curve p value: Probability value CI: Confidence Intervals.

NPV: Negative predictive value PPV: Positive predictive value.

*: Statistically significant at *p* ≤ 0.05 #Cut off was chosen according to Youden index.

Handbreadth, index and ring proximal phalangeal lengths, and little total length had significant p-values (*p* ≤ 0.05), while thumb proximal phalanx did not, indicating weaker association with ethnicity.

Index proximal phalangeal length showed 85.0% sensitivity at ≤ 2.6 cut-off. Specificity varied, with hand length showing lower values.

Optimal cut-offs were handbreadth > 9.3, index proximal phalangeal length ≤ 2.6. Accuracy ranged from 57.5% to 68.75%, with higher rates for hand length and index proximal phalangeal length.

**Fig. 3:**
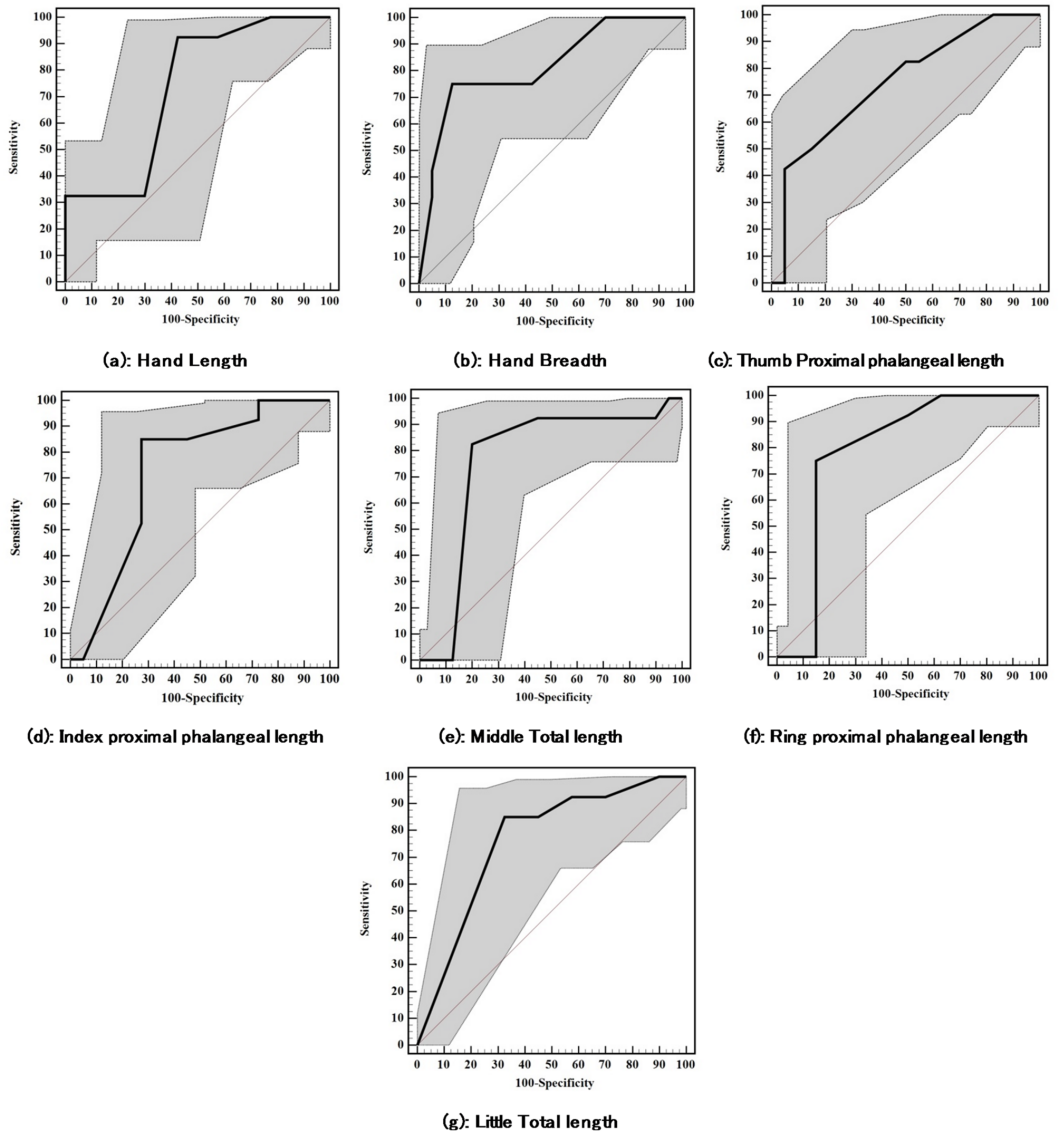
ROC curves for different measures to distinguish Saudis from Egyptians in female participants.

In females (Table 9; Fig. 3), all measurements proved to be significant discriminators (*p* < 0.001), handbreadth (AUC = 0.818; 95% CI: 0.724–0.912) had the strongest discriminatory power, followed by ring proximal phalanx (AUC = 0.788) and little total length (AUC = 0.763). Hand length (AUC = 0.732) and thumb proximal phalanx (AUC = 0.745) showed moderate discrimination.

**Table 9:** Diagnostic performance for different measures to distinguish Saudis from Egyptians in female participants.

		AUC	*p*	95% C. I	Cut off	Sensitivity	Specificity	PPV	NPV	Accuracy
Hand	Length	0.732	< 0.001^*^	0.620–0.844	≤ 17^#^	92.50	57.50	68.5	88.5	75.0
	Breadth	0.818	< 0.001^*^	0.724–0.912	≤ 7.8	75.0	57.50	63.8	69.7	66.25
Thumb	Proximal phalangeal length	0.745	< 0.001^*^	0.637–0.853	≤ 3	82.50	50.0	62.3	74.1	66.25
Index	Proximal phalangeal length	0.727	< 0.001^*^	0.609–0.844	≤ 2.2^#^	85.0	72.50	75.6	82.9	78.75
Middle	Total length	0.764	< 0.001^*^	0.645–0.884	≤ 7.5	92.50	55.0	67.3	88.0	73.75
Ring	Proximal phalangeal length	0.788	< 0.001^*^	0.676–0.901	≤ 2	92.50	50.0	64.9	87.0	71.25
Little	Total length	0.763	< 0.001^*^	0.656–0.871	≤ 5^#^	85.0	67.50	72.3	81.8	76.25

AUC: Area Under a Curve p value: Probability value CI: Confidence Intervals.

NPV: Negative predictive value PPV: Positive predictive value.

*: Statistically significant at *p* ≤ 0.05 #Cut off was chosen according to Youden index.

All measures were statistically significant (*p* < 0.001) with narrow confidence intervals, indicating reliable group differentiation.

Hand length had highest sensitivity (92.5%, ≤ 17 cut-off). Index proximal phalanx balanced sensitivity (85.0%) and specificity (72.5%), making it a strong overall performer.

Optimal cut-offs for females were handbreadth ≤ 7.8, index proximal phalanx ≤ 2.2. Accuracy ranged from 66.25% to 78.75%, highest for index proximal phalanx, followed by little total length, indicating moderate reliability.

The DeLong method was employed to determine the 95% CIs for all AUC values, accounting for sampling variability within each sex group.

## Discussion

Hands, the most valuable assets of the body, are the subject of a comprehensive study known as hand anthropometry. This systematic measurement of hand size, shape, and proportion provides a wealth of information about the development of the hand, and its relationship to age, sex, ethnicity, body mass index, nutritional status, and sedentary comportment. Also, anthropometric data on hand dimensions have been used in resolving legal, medical, and forensic cases^4,6^.

Establishing the biological profile of unknown individuals comprises identifying the person’s age, sex, ethnicity, and stature. Body weight, a fundamental aspect of human biology, is crucial in identifying individuals in forensic investigations. It is also used to assess growth patterns and adjust therapeutic dosages, demonstrating its versatility and importance in various fields. However, its significance is most pronounced in clinical examinations, where body weight measurement can be challenging due to illness and body deformity, making it an important tool in forensic identification^8,9,14^.

Many earlier studies demonstrated the relationship between the anthropometric dimensions of the foot and body weight^38–40^. In addition, El-Meligy et al.^40^ developed a regression equation using tibial length and bimalleolar breadth to predict the deceased’s weight.

On the other hand, while previous studies have demonstrated the use of palm and hand measurements for sex and stature determination, with implications for person identification, our literature review revealed a scarcity of research on body weight estimation from hand anthropometry^41–43^. Our study aims to predict body weight and ethnicity based on hand and finger dimensions in Egyptian and Saudi populations.

### Methodological considerations

The validity of anthropometric prediction models is fundamentally dependent on the precision of the underlying measurements. In the present study, reliability metrics demonstrated exceptional consistency, with the majority of inter-rater and intra-rater ICCs exceeding 0.95. Notably, several phalangeal segments achieved an ICC of 1.000, indicating that the landmarks used for measurement were clearly defined and reproducible across observers. The low TEM values further confirm that measurement error was negligible relative to the biological variance of the sample. By minimizing the contribution of observer-induced error to the total variance, we have significantly reduced the risk of Type I errors in our regression models. This high level of precision strengthens the clinical and forensic utility of our equations, particularly the Saudi female “Little finger” model, which demonstrated the highest predictive accuracy supported by excellent reliability.

### Main findings and comparison with previous studies

The results of Egyptian body weight in the current study are comparable to those reported by Hussein et al.^44^. At the same time, the present study showed that the Egyptian population was non-significantly fatter than the Saudi population. Several studies have demonstrated variation in body weight across populations. Differences in ethnic and population-genetic backgrounds can explain this^45–48^.

Moreover, the current study demonstrated significantly higher hand and finger measurements in males than in females in both groups. This result confirmed those of many previous studies conducted in Egypt, Saudi Arabia, and other countries worldwide^32,44,49–59^.

Ernsten’s research elucidates that the basis of sexual diversity is deeply rooted in the complex relations of biology, genetics, and social and physical environments. Genetics, as Ernsten’s work emphasizes, is the primary determinant of the gonads in offspring, with sex hormones like androgens and estrogens playing a secondary role in promoting the phenotypic differentiation of males and females^60^. One notable phenotypic difference between male and female humans is the hands, with males generally having larger hands than females. Therefore, the hand could have a potential value for sex estimation^61^.

The present study demonstrated comparable hand measurements (hand length, handbreadth, and palm length) in the Egyptian population to those reported in previous studies of Egyptians^32,44,52^. Moreover, hand measurements of the participating Saudis in the present study were consistent with those reported in previous Saudi research^54,55,62^.

When comparing the two studied populations by hand measurements, the current study found that Egyptians had non-significantly longer hands than Saudis. Moreover, Saudi males had broader hands than Egyptian males, a result that aligns with that of Foad et al.^49^. However, Saudi females had narrower hands than Egyptian females, a finding that contrasts with that of Foad et al.^49^ and Supare MS^63^.

Anthropometric measurements have been observed for years to differ not only among countries but also among different ethnicities within countries^43^. Our comprehensive study, when compared with other populations, revealed intriguing findings. Egyptian and Saudi males had longer, narrower hands than Indian males, and longer, wider hands than Bangladeshi, Turkish, Nigerian, and Sabahan males. In contrast, Egyptian females had longer and narrower hands than Indian females, and longer and broader hands than Bangladeshi and Turkish females, shorter and wider than Nigerian females. Interestingly, Saudi females had hand lengths and widths comparable to those of Indian, Bangladeshi, Turkish, and Nigerian females^64–67^.

The anthropometric variations identified in the Egyptian and Saudi cohorts are consistent with broader international patterns described by Sahin et al. (2024), who reported significant geographic and ethnic differences in hand morphometry. Specifically, Egyptian and Saudi male cohorts exhibited hand lengths greater than those documented for Turkish, Moroccan, and Gabonese males, and similar to those observed in Syrian and Senegalese males. Additionally, the observed differences in handbreadth between Egyptian (8.02 cm) and Saudi (7.99 cm) female cohorts mirror the international diversity reported by Sahin et al., who found female hand width ranged from approximately 7.97 cm in Turkey to 8.36 cm in Senegal. Sahin et al. further classified hand types into four distinct categories (Type 1: Wide hand, long finger; Type 2: Long palm and finger; Type 3: Narrow hand, short finger; Type 4: Short palm, short finger), noting that Moroccan and Kazakhstan populations were predominantly characterized by Type 3 or Type 4 hand types. This international context supports the application of ROC curve analysis to differentiate between Egyptian and Saudi ethnicities, reinforcing the notion that hand morphometry is a reliable indicator of geographic and ethnic origin^68^.

Regarding individual finger measurements, the results of the present study are consistent with previous Egyptian and Saudi studies^32,53,56,62^. At the same time, the present study’s results were higher than those reported in studies of other populations.

Our objective comparison of the two studied populations, the Egyptians and Saudis, has revealed some interesting findings. Egyptian males, for instance, showed non-significantly higher values of total and proximal phalangeal length of the thumb than the Saudi males. Similarly, Egyptian females exhibited larger total thumb length. In addition, some individual finger and phalange measurements were higher in Egyptian males, and others were higher in Saudi males. While Egyptian females consistently have higher values of individual finger measurements than Saudi females, except for the distal phalangeal length of the little finger.

In their study, Bhat et al.^13^ concluded that there were marked differences in hand length and individual finger measurements across the seven population groups studied: Indians, Malaysians, Chinese, Dutch, Africans, Iranians, and Poles.

Moreover, Davies et al. compared hand dimensions among Black, Indian, and Caucasian residents of the West Indies^20^. They concluded that Black individuals had larger hand dimensions than both Caucasians and Indians, who had comparable hand measurements. On the other hand, Courtney’s study^21^ focused on Hong Kong Chinese women and concluded that they had smaller hands than those of the British and Americans, but larger than those of the Japanese.

Imrhan’s study^69^, which involved 50 female Indians, primarily compared the means of the measured right-hand dimensions with those of similar measurements in females from Hong Kong, Japan, the U.K., and the U.S.A. (Air Force and Vietnamese subpopulations). His conclusion that most hand dimensions in Indian females were significantly smaller than those of Hong Kong, U.S. Air Force, and U.K. females, with Vietnamese Americans and Japanese showing the least differences from Indians, is the study’s key finding.

However, Khazri et al.^67^ found no significant difference among the four studied ethnicities of Sabah, namely, Kadazandusun, Bajau, Malay, and Chinese populations in their hand measurements.

Our study, which focused on predicting body weight from hand measurements, has direct and practical implications for anthropometry and body measurement studies. By performing univariate linear regression analysis to predict body weight from all the studied hand and finger measurements, we have provided a practical tool for researchers and anthropometrists.

In Egyptian males, the Handbreadth has a positive coefficient (+ 14.73), while Hand Length and Index Distal have negative coefficients. This suggests that, among Egyptian males, a wider but shorter hand is a stronger indicator of higher body mass in this sample.

The distal and proximal phalangeal lengths of the index finger were the most reliable indicators for body weight prediction in female Egyptians, with correlation coefficients of 0.572 and 0.513, respectively.

In the Saudi male group, handbreadth remains the strongest individual predictor of body weight. Index Middle Phalanx has a substantial impact on the equation (B = 60.932), suggesting that for every 1 centimeter increase in this bone, the predicted weight increases significantly.

In the Saudi female population, the total lengths of the middle, little, and ring fingers were the best determinants of body weight, showing a very strong positive linear relationship. These results surpass those reported by Nataraja et al.^45^, which ranged from 0.110 to 0.367. At the same time, in their study of the Ghanaian population, Okwan et al.^70^ concluded that hand length and breadth best estimate body weight.

Furthermore, the present study developed multiple regression equations from different hand measurements in males and females of the two studied populations. This improves the accuracy for body weight determination, as shown by higher correlation coefficients and a strong to very strong relationship. Despite the inherent anatomical correlation between various hand and finger dimensions, our multivariate models demonstrated robust stability. The VIF values across all finalized equations remained consistently low (ranging from 1.017 to 2.680), well within acceptable limits to rule out significant multicollinearity. For instance, the high predictive power observed in the Saudi female “Little finger” model (R2 = 0.817) was achieved with VIF values near 1.3, confirming that each phalangeal segment provided unique, non-redundant information for predicting body weight. Consequently, the residual variance explained by these models reflects genuine biological association rather than statistical inflation.

The second aim of our study is to predict ethnicity from hand and finger measurements. We designed Receiver Operating Characteristic curves for the most significantly different hand measurements between the two studied groups to predict ethnicity in male and female participants separately. The AUC values for ethnic and sex discrimination demonstrated high accuracy; however, the associated 95% Confidence Intervals (CIs) reflect inherent sampling variability. The wider intervals observed in the male group are due to the specific sample size (40 per group), which increases the margin of error compared to larger datasets. Despite this, the lower bounds of the CIs remain statistically significant (above 0.5), supporting the validity of these anthropometric parameters as discriminatory tools. Hand measurements in females were highly “ethnicity-specific,” likely due to more distinct sexual dimorphism patterns or genetic growth factors between the two populations.

However, it is crucial to keep in mind that individuals from different parts of the world have distinct morphological traits based on their ethnic and geographic locations. This diversity makes it impossible for a single formula to accurately reflect conditions worldwide. Therefore, it is recommended that comparable research be conducted across multiple populations.

### Practical implications

Our study provides practical regression tools for estimating body weight from hand and finger measurements in Egyptian and Saudi populations. These equations offer valuable applications in settings where direct measurement of body weight is impractical or impossible, such as in medico-legal investigations, archaeological assessments, or clinical situations involving immobilized or deceased individuals. By enabling body weight estimation from accessible hand measurements, our findings can support identity verification, nutritional assessment, and individualized medical dosing.

### Limitations and future research

A key limitation of our study is the restricted generalizability of the predictive equations, as morphological traits and hand dimensions vary widely across global populations. The models are most accurate when applied to populations similar to those studied; caution is warranted when extending their use to other ethnic or demographic groups. Additionally, the sample size was relatively modest, which may have affected the precision of our estimates. Future research should validate and refine these models in larger and more diverse populations to establish broader applicability and utility.

Moreover, despite these robust correlations, certain limitations must be acknowledged to avoid overgeneralization. The regression models derived here are specific to the young adult age group (mean age 23) and may not accurately reflect pediatric or geriatric populations where bone density and soft tissue proportions differ. Additionally, the study did not account for external variables such as manual labor intensity or nutritional status, which can influence handbreadth and phalangeal proportions.

Furthermore, Due to the sampling variability reflected in the wider Confidence Intervals of the AUC, we recommend future studies with larger cohorts to refine these intervals and enhance the precision of ethnic discrimination models.

Ultimately, while this research underscores the potential of hand measurements as morphological proxies, these models should be considered population-specific. We recommend further large-scale, multi-center research involving diverse age groups and socio-economic backgrounds to validate these anthropometric techniques and enhance their global forensic utility.

## Conclusion

The present study establishes hand and finger anthropometry as a practical, non-invasive, and statistically significant tool for estimating body weight and identifying ethnicity within forensic and clinical contexts. Our findings confirm a high degree of sexual dimorphism across both Egyptian and Saudi cohorts, emphasizing that sex-specific models are essential for accurate identification. Morphologically, Egyptian participants generally exhibited larger hand dimensions than the Saudi cohort, particularly in hand length and total thumb dimensions among females. Regarding the predictive power of these measurements, population-specific variations were evident. In Egyptian males, the middle phalanx of the index finger and handbreadth were the most reliable determinants of body weight, whereas the distal phalanx of the index finger was the primary predictor for Egyptian females. Conversely, Saudi male weight was best predicted by handbreadth and the middle phalanx of the index, while Saudi females showed an exceptionally high correlation with the total length of the middle and little fingers. For ethnic discrimination, the proximal phalanx of the index finger (in males) and handbreadth (in females) emerged as the most potent discriminatory parameters, achieving significant accuracy in ROC curve analysis. Interestingly, the hand measurements in females are much better at distinguishing ethnicity than in males.

## Electronic Supplementary Material

Below is the link to the electronic supplementary material.

Supplementary Material 1.

Supplementary Material 2.

## Data Availability

The authors confirm that the data supporting the findings are available from the corresponding author upon reasonable request. Data analysis is provided within the manuscript.
