# Supplementary material for: Prediction of body weight and ethnicity using anthropomorphic measurements of the hand in two different populations
Source: Sci Rep. 2026 Apr 4;16:11609. doi: 10.1038/s41598-026-43161-z (PMC13056951; doi:10.1038/s41598-026-43161-z)
Supplement: Supplementary file 1 — Supplementary Material 1. [file 41598_2026_43161_MOESM1_ESM.docx]

**Supplementary table S1: Matrix correlation between hand and fingers’ measurements in Egyptian males (n = 40)**

|  | **BW** | **Hand. L** | **Hand. B** | **Index. T** | **Index. D** | **Index. M** | **Middle. T** | **Middle. M** | **Ring. M** |
| --- | --- | --- | --- | --- | --- | --- | --- | --- | --- |
| **BW** | 1.000 |  |  |  |  |  |  |  |  |
| **Hand. L** | -0.424 | 1.000 |  |  |  |  |  |  |  |
| **Hand. B** | 0.519 | 0.141 | 1.000 |  |  |  |  |  |  |
| **Index. T** | -0.508 | 0.694 | -0.038 | 1.000 |  |  |  |  |  |
| **Index. D** | -0.390 | -0.079 | -0.172 | 0.384 | 1.000 |  |  |  |  |
| **Index. M** | -0.552 | 0.796 | -0.010 | 0.835 | -0.030 | 1.000 |  |  |  |
| **Middle. T** | -0.356 | 0.624 | 0.159 | 0.593 | 0.262 | 0.475 | 1.000 |  |  |
| **Middle. M** | -0.396 | 0.288 | -0.285 | 0.391 | 0.497 | 0.158 | 0.780 | 1.000 |  |
| **Ring. M** | 0.411 | -0.813 | 0.047 | -0.765 | -0.056 | -0.809 | -0.665 | -0.509 | 1.000 |

Data presented in Pearson coefficient (r)

**Supplementary table S2: Matrix correlation between hand and fingers’ measurements in Egyptian females (n=40)**

|  | **Body weight** | **Index** Distal phalangeal length | **Index** Proximal phalangeal length | **Middle**  Total length | **Middle** Distal phalangeal length |
| --- | --- | --- | --- | --- | --- |
| **Body weight** | 1.000 |  |  |  |  |
| **Index** Distal phalangeal length | 0.572^*^ | 1.000 |  |  |  |
| **Index** Proximal phalangeal length | -0.513^*^ | -0.272 | 1.000 |  |  |
| **Middle** Total length | 0.376^*^ | 0.429^*^ | 0.026 | 1.000 |  |
| **Middle** Distal phalangeal length | 0.366^*^ | 0.343^*^ | -0.020 | 0.555^*^ | 1.000 |

Data presented in Pearson coefficient (r)

**Supplementary table S3: Matrix correlation between hand and fingers’ measurements in Saudi males (n = 40)**

|  | **Body weight** | **Hand** Breadth | **Thumb** Distal phalangeal length | **Index** Total length | **Index** Middle phalangeal length | **Middle** Total length |
| --- | --- | --- | --- | --- | --- | --- |
| **Body weight** | 1.000 |  |  |  |  |  |
| **Hand** Breadth | 0.621^*^ | 1.000 |  |  |  |  |
| **Thumb** Distal phalangeal length | 0.404^*^ | 0.401^*^ | 1.000 |  |  |  |
| **Index** Total length | 0.320^*^ | 0.551^*^ | 0.685^*^ | 1.000 |  |  |
| **Index** Middle phalangeal length | 0.533^*^ | 0.437^*^ | 0.475^*^ | 0.623^*^ | 1.000 |  |
| **Middle** Total length | 0.329^*^ | 0.498^*^ | 0.708^*^ | 0.904^*^ | 0.638^*^ | 1.000 |

Data presented in Pearson coefficient (r)

**Supplementary table S4a: Matrix correlation between hand and fingers’ measurements in Saudi Females (n = 40)**

|  | **Body weight** | **Hand** Length | **Hand** Breadth |
| --- | --- | --- | --- |
| **Body weight** | 1.000 |  |  |
| **Hand** Length | 0.781^*^ | 1.000 |  |
| **Hand** Breadth | 0.593^*^ | 0.858^*^ | 1.000 |

Data presented in Pearson coefficient (r)

**Supplementary table S4b: Matrix correlation between hand and fingers’ measurements in Saudi Females (n = 40)**

|  | **Body weight** | **Thumb** Distal phalangeal length | **Thumb** Proximal phalangeal length |
| --- | --- | --- | --- |
| **Body weight** | 1.000 |  |  |
| **Thumb** Distal phalangeal length | -0.353^*^ | 1.000 |  |
| **Thumb** Proximal phalangeal length | 0.629^*^ | -0.372^*^ | 1.000 |

Data presented in Pearson coefficient (r)

**Supplementary table S4c: Matrix correlation between hand and fingers’ measurements in Saudi Females (n = 40)**

|  | **Body weight** | **Index Total length** | **Index Middle phalangeal length** | **Index Proximal phalangeal length** |
| --- | --- | --- | --- | --- |
| **Body weight** | 1.000 |  |  |  |
| **Index Total length** | 0.774^*^ | 1.000 |  |  |
| **Index Middle phalangeal length** | 0.695^*^ | 0.879^*^ | 1.000 |  |
| **Index Proximal phalangeal length** | 0.549^*^ | 0.512^*^ | 0.263 | 1.000 |

Data presented in Pearson coefficient (r)

**Supplementary table S4d: Matrix correlation between hand and fingers’ measurements in Saudi Females (n = 40)**

|  | **Body weight** | **Middle Distal phalangeal length** | **Middle Middle phalangeal length** | **Middle Proximal phalangeal length** |
| --- | --- | --- | --- | --- |
| **Body weight** | 1.000 |  |  |  |
| **Middle Distal phalangeal length** | 0.438^*^ | 1.000 |  |  |
| **Middle Middle phalangeal length** | 0.742^*^ | -0.191 | 1.000 |  |
| **Middle Proximal phalangeal length** | 0.595^*^ | 0.179 | 0.338^*^ | 1.000 |

Data presented in Pearson coefficient (r)

**Supplementary table S4e: Matrix correlation between hand and fingers’ measurements in Saudi Females (n = 40)**

|  | **Body weight** | **Ring** Distal phalangeal length | **Ring** Middle phalangeal length | **Ring** Proximal phalangeal length |
| --- | --- | --- | --- | --- |
| **Body weight** | 1.000 |  |  |  |
| **Ring** Distal phalangeal length | 0.615^*^ | 1.000 |  |  |
| **Ring** Middle phalangeal length | 0.681^*^ | 0.130 | 1.000 |  |
| **Ring** Proximal phalangeal length | 0.721^*^ | 0.344^*^ | 0.721^*^ | 1.000 |

Data presented in Pearson coefficient (r)

**Supplementary table S4f: Matrix correlation between hand and fingers’ measurements in Saudi Females (n = 40)**

|  | **Body weight** | **Little** Distal phalangeal length | **Little** Middle phalangeal length | **Little** Proximal phalangeal length |
| --- | --- | --- | --- | --- |
| **Body weight** | 1.000 |  |  |  |
| **Little** Distal phalangeal length | 0.419^*^ | 1.000 |  |  |
| **Little** Middle phalangeal length | 0.362^*^ | 0.166 | 1.000 |  |
| **Little** Proximal phalangeal length | 0.404^*^ | -0.440^*^ | -0.278 | 1.000 |

Data presented in Pearson coefficient (r)
