# Supplementary material for: Prediction of body weight and ethnicity using anthropomorphic measurements of the hand in two different populations
Source: Sci Rep. 2026 Apr 4;16:11609. doi: 10.1038/s41598-026-43161-z (PMC13056951; doi:10.1038/s41598-026-43161-z)
Supplement: Supplementary file 2 — Supplementary Material 2. [file 41598_2026_43161_MOESM2_ESM.docx]

**
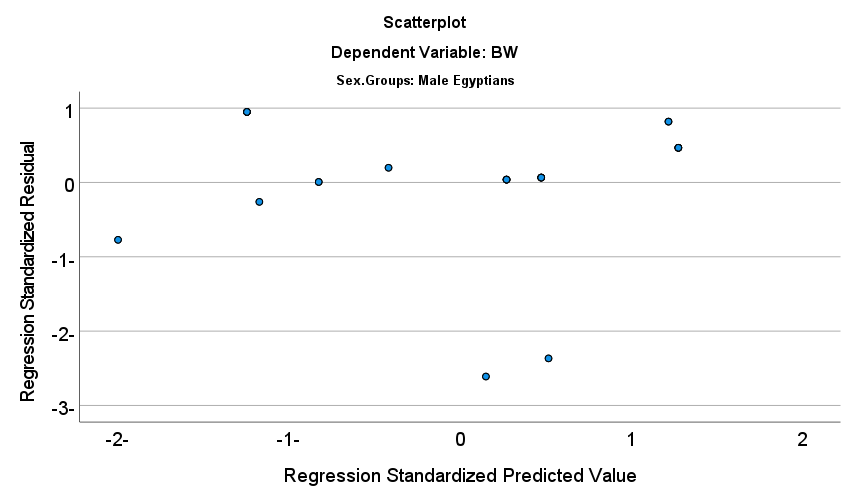
**

**Supplementary Figure S1: Checks for homoscedasticity (Residual Plot) in Egyptian Males.**

**
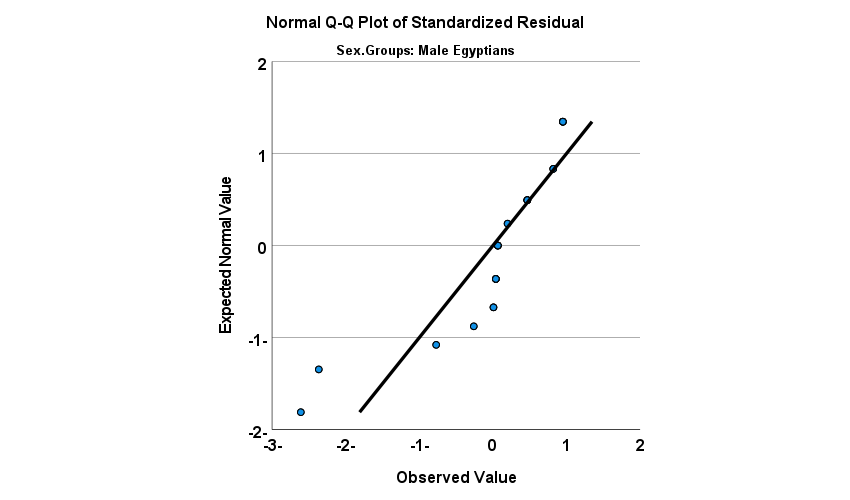
**

**Supplementary Figure S2: Normality of Residuals (Q-Q Plot) in Egyptian males. Maximum Cook's Distance = 0.539, R^2^=0.634; % variance unexplained (100-R²) = 36.6%.**

**
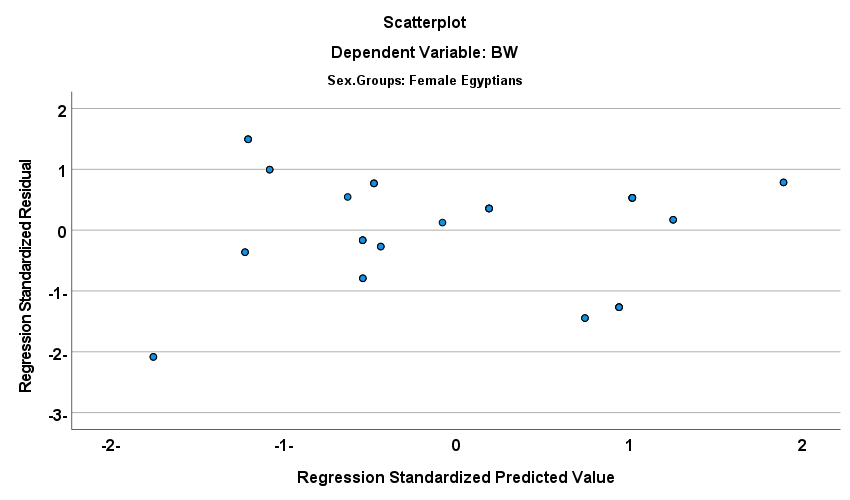
**

**Supplementary Figure S3: Checks for homoscedasticity (Residual Plot) in Egyptian Females.**

**
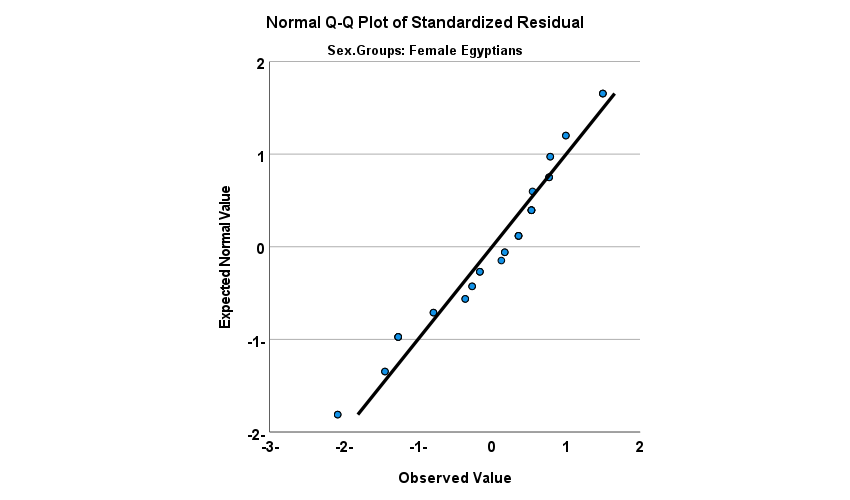
**

**Supplementary Figure S4: Normality of Residuals (Q-Q Plot) in Egyptian males. Maximum Cook's Distance = 0.176, R^2^=0.525; % variance unexplained (100-R²) = 47.5%.**

**
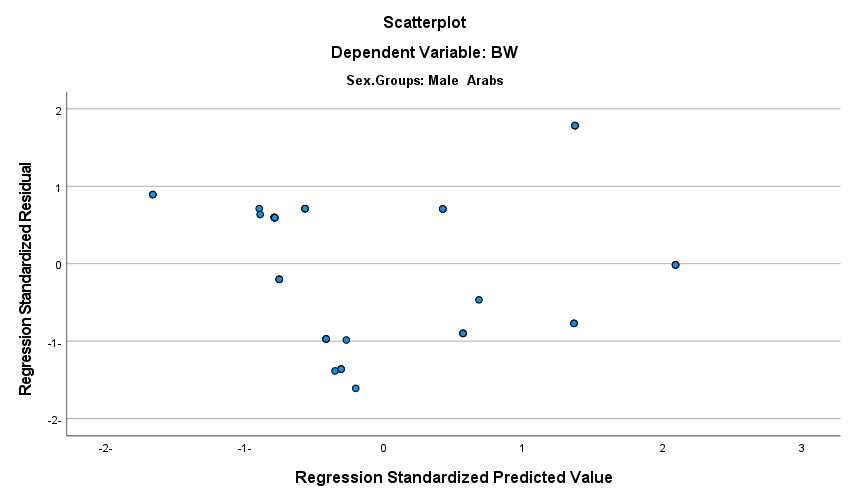
**

**Supplementary Figure S5: Checks for homoscedasticity (Residual Plot) in Saudi Males.**

**
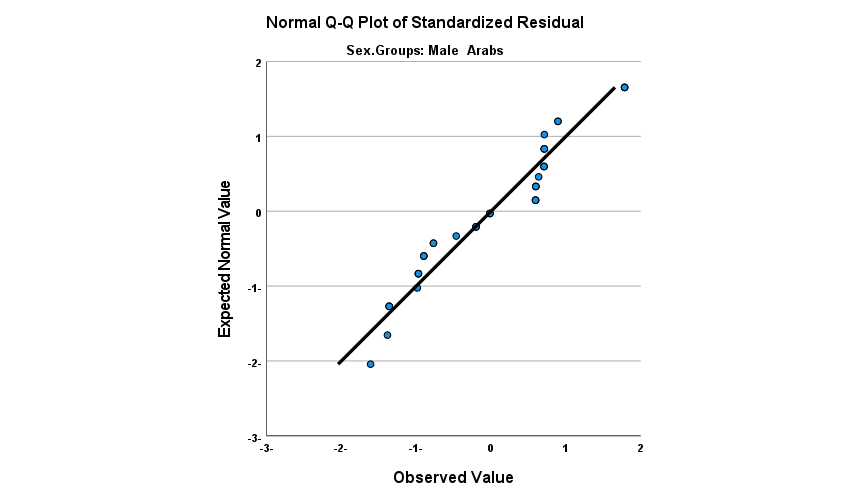
**

**Supplementary Figure S6: Normality of Residuals (Q-Q Plot) in Saudi males. Maximum Cook's Distance = 0.087, R^2^=0.553; % variance unexplained (100-R²) = 44.7%.**

**
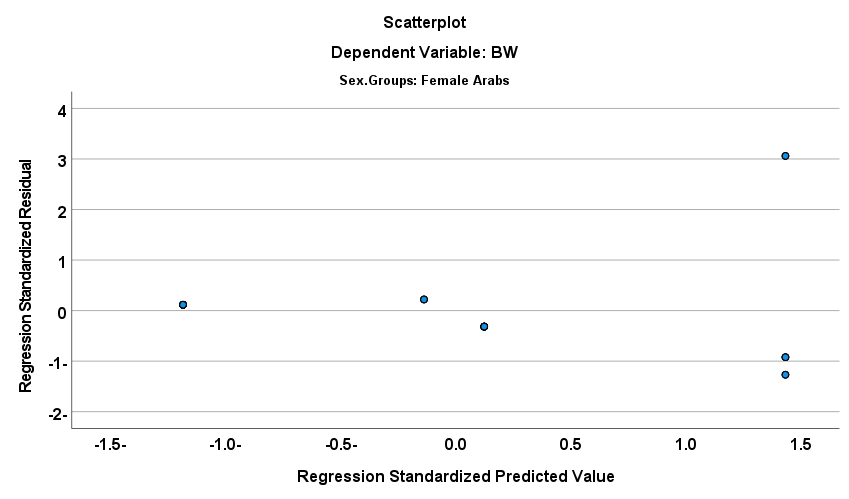
**

**Supplementary Figure S7a1: Checks for homoscedasticity (Residual Plot) for the body weight, hand length and hand breadth in Saudi females.**

**
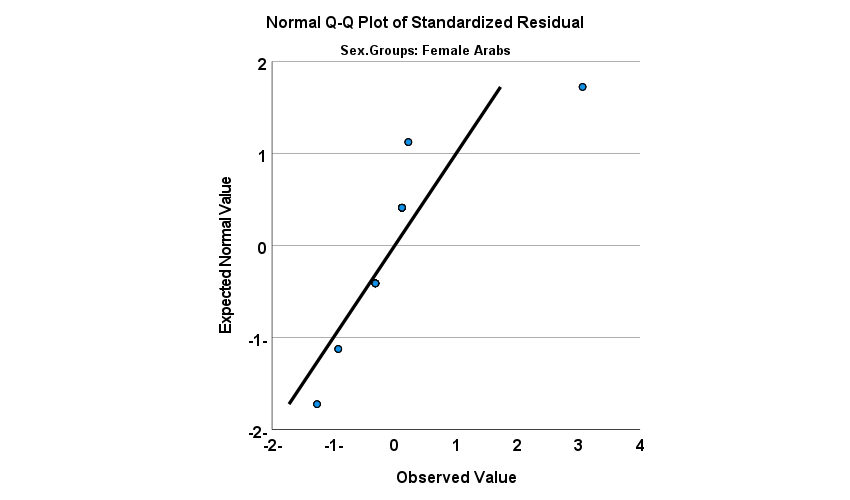
**

**Supplementary Figure S7a2: Normality of Residuals (Q-Q Plot) for the body weight, hand length and hand breadth) in Saudi females. Maximum Cook's Distance = 0.427, R^2^=0.352; % variance unexplained (100-R²) = 64.8%.**

**
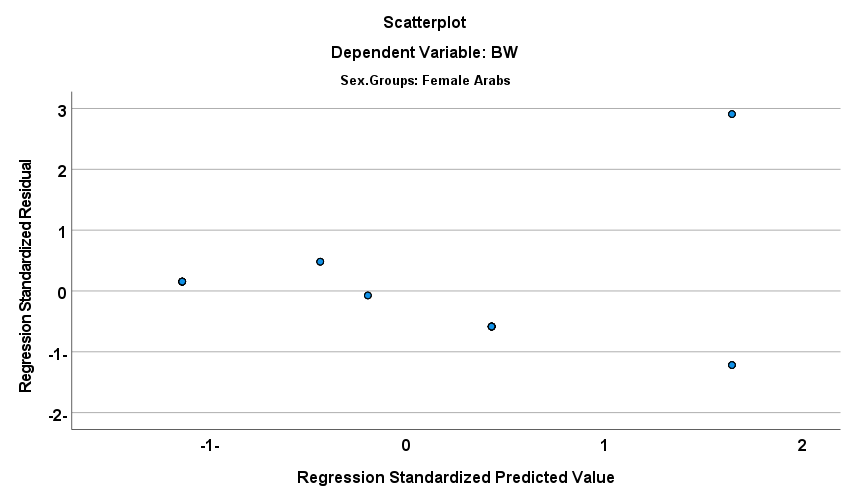
**

**Supplementary Figure S7b1: Checks for homoscedasticity (Residual Plot) for the body weight, thumb distal phalangeal length, thumb proximal phalangeal length in Saudi females.**

**
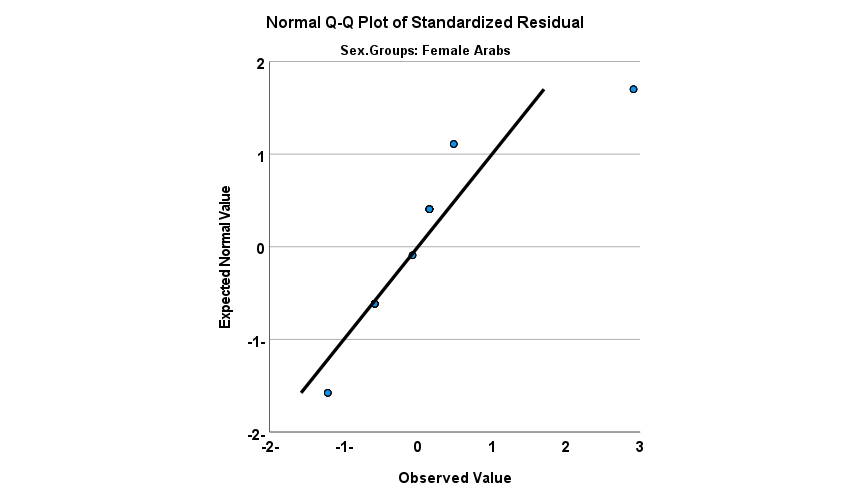
**

**Supplementary Figure S7b2: Normality of Residuals (Q-Q Plot) for the body weight, thumb distal phalangeal length, thumb proximal phalangeal length in Saudi females. Maximum Cook's Distance = 0.363, R^2^=0.412; % variance unexplained (100-R²) = 58.8%.**

**
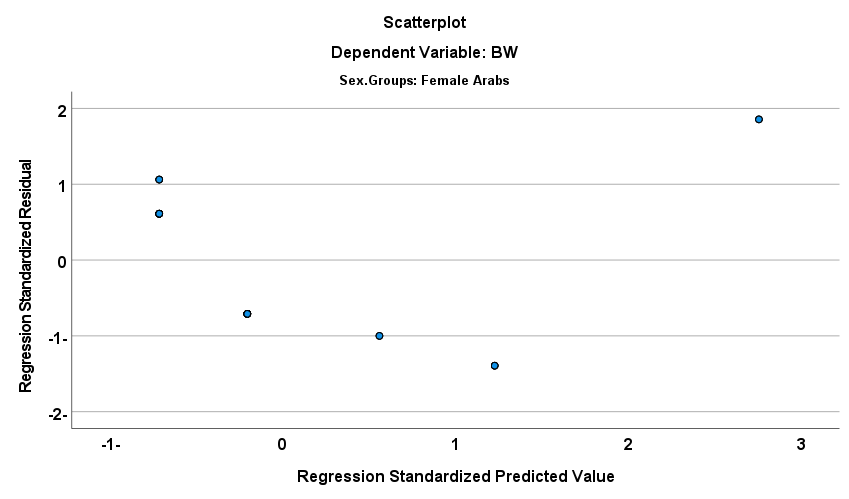
**

**Supplementary Figure S7c1: Checks for homoscedasticity (Residual Plot) for the Body weight, index total length, index middle phalangeal length, index proximal phalangeal length in Saudi females.**

**
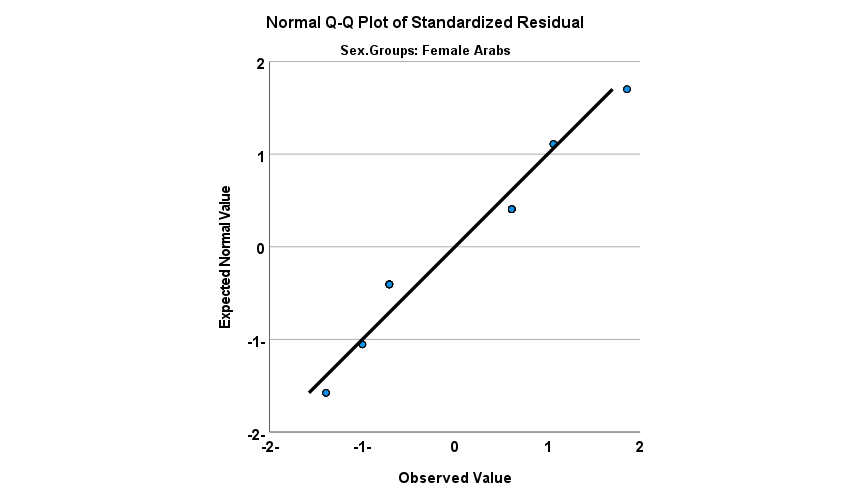
**

**Supplementary Figure S7c2: Normality of Residuals (Q-Q Plot) for the body weight, index total length, index middle phalangeal length, index proximal phalangeal length in Saudi females. Maximum Cook's Distance = 0.440, R^2^=0.627; % variance unexplained (100-R²) = 37.3%.**

**
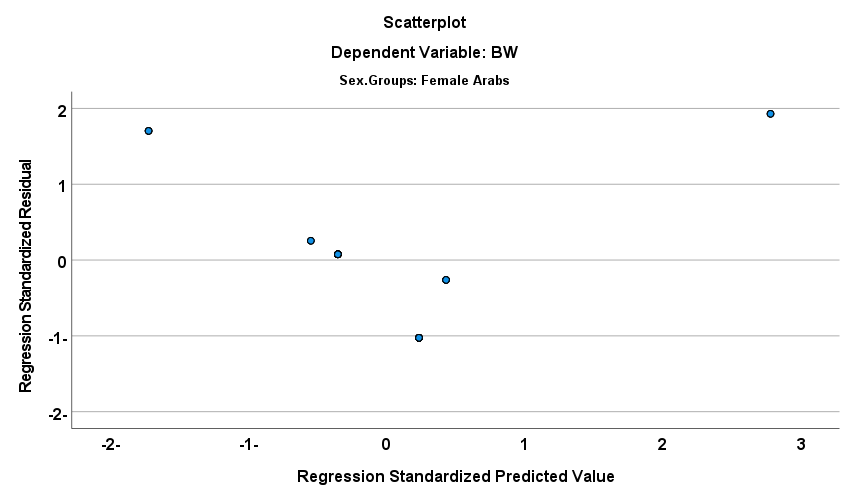
**

**Supplementary Figure S7d1: Checks for homoscedasticity (Residual Plot) for the body weight, middle distal phalangeal length, middle middle phalangeal length, middle proximal phalangeal length in Saudi females.**

**
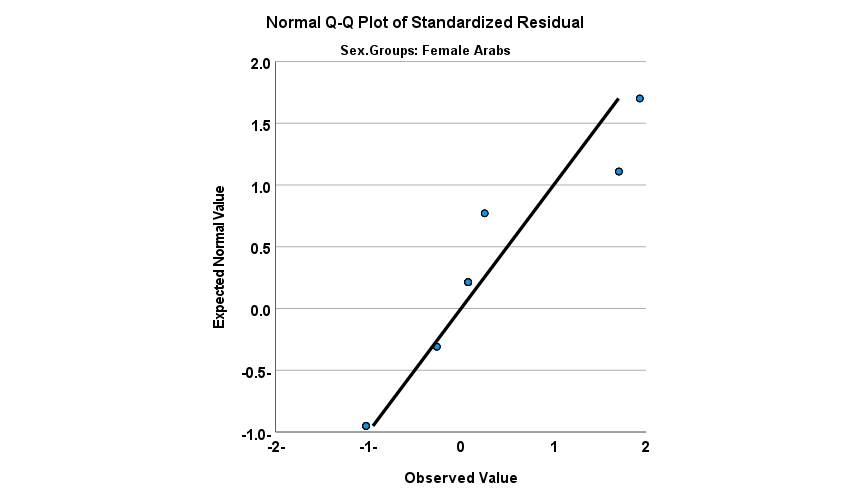
**

**Supplementary Figure S7d2: Normality of Residuals (Q-Q Plot) for the body weight, middle distal phalangeal length, middle middle phalangeal length, middle proximal phalangeal length in Saudi females. Maximum Cook's Distance = 0.440, R^2^=0.627; % variance unexplained (100-R²) = 37.3%, Maximum Cook's Distance = 0.468, R^2^=0.468; % variance unexplained (100-R²) = 53.2%.**

**
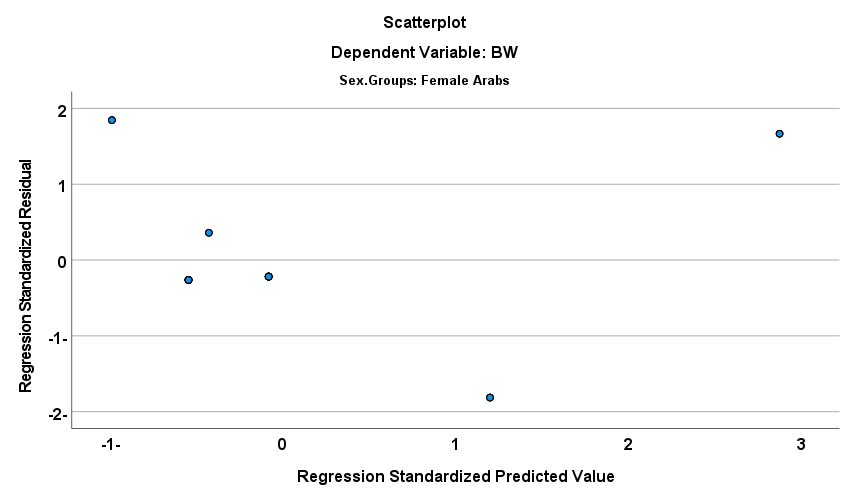
**

**Supplementary Figure S7e1: Checks for homoscedasticity (Residual Plot) for the body weight, ring distal phalangeal length, ring middle phalangeal length, ring proximal phalangeal length in Saudi females.**

**
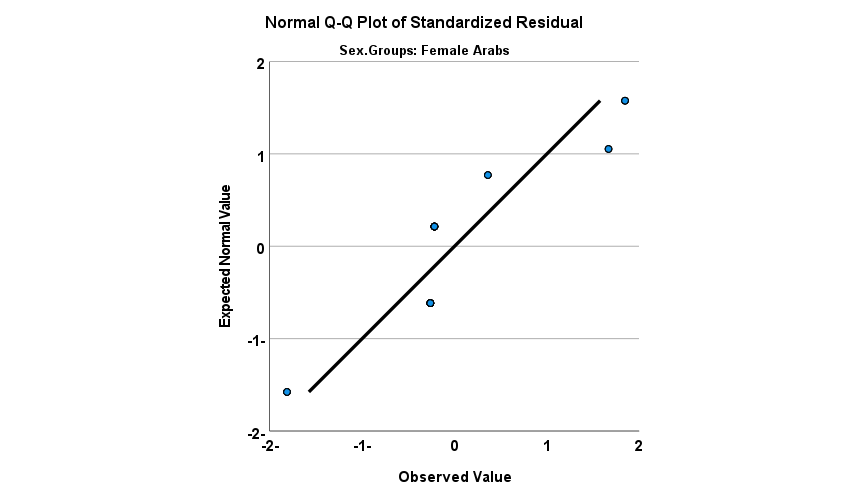
**

**Supplementary Figure S7e2: Normality of Residuals (Q-Q Plot) for the body weight, ring distal phalangeal length, ring middle phalangeal length, ring proximal phalangeal length in Saudi females. Maximum Cook's Distance = 0.387, R^2^=0.746; % variance unexplained (100-R²) = 25.4%.**

**
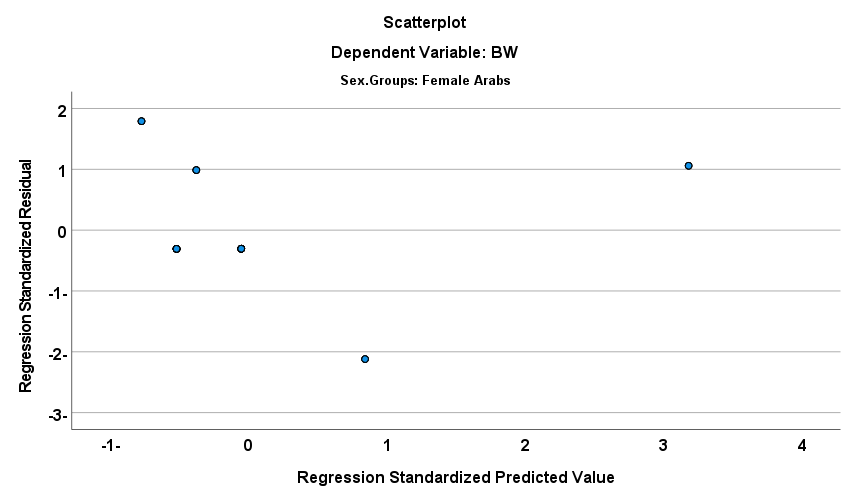
**

**Supplementary Figure S7f1: Checks for homoscedasticity (Residual Plot) for the body weight, little distal phalangeal length, little middle phalangeal length, little proximal phalangeal length in Saudi females.**

**
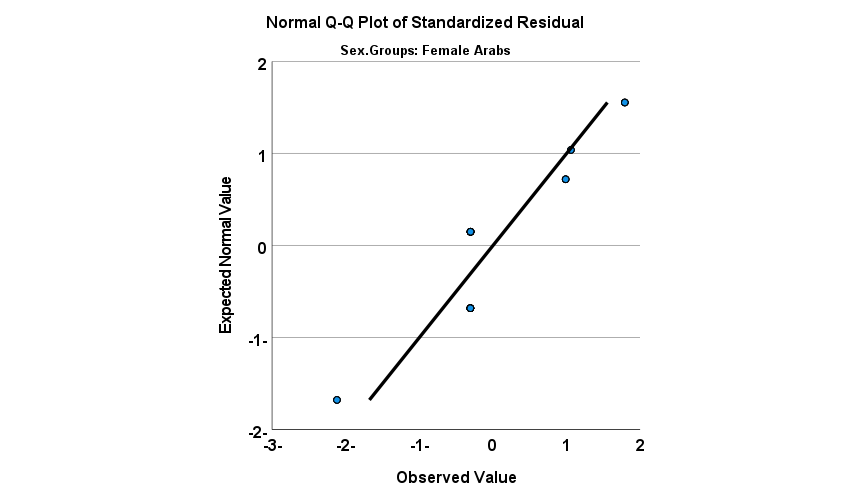
**

**Supplementary Figure S7f2: Normality of Residuals (Q-Q Plot) for the body weight, little distal phalangeal length, little middle phalangeal length, little proximal phalangeal length in Saudi females. Maximum Cook's Distance = 0.374, R^2^=0.817; % variance unexplained (100-R²) = 18.3%.**
